# Overview of biofertilizers in crop production and stress management for sustainable agriculture

**DOI:** 10.3389/fpls.2022.930340

**Published:** 2022-08-23

**Authors:** Parul Chaudhary, Shivani Singh, Anuj Chaudhary, Anita Sharma, Govind Kumar

**Affiliations:** ^1^Govind Ballabh Pant University of Agriculture and Technology, Pantnagar, India; ^2^School of Agriculture and Environmental Science, Shobhit University, Gangoh, India; ^3^Department of Crop Production, Central Institute for Subtropical Horticulture, Lucknow, India

**Keywords:** abiotic stress, biotic stress, biofertilizers, crop productivity, plant-root interaction

## Abstract

With the increase in world population, the demography of humans is estimated to be exceeded and it has become a major challenge to provide an adequate amount of food, feed, and agricultural products majorly in developing countries. The use of chemical fertilizers causes the plant to grow efficiently and rapidly to meet the food demand. The drawbacks of using a higher quantity of chemical or synthetic fertilizers are environmental pollution, persistent changes in the soil ecology, physiochemical composition, decreasing agricultural productivity and cause several health hazards. Climatic factors are responsible for enhancing abiotic stress on crops, resulting in reduced agricultural productivity. There are various types of abiotic and biotic stress factors like soil salinity, drought, wind, improper temperature, heavy metals, waterlogging, and different weeds and phytopathogens like bacteria, viruses, fungi, and nematodes which attack plants, reducing crop productivity and quality. There is a shift toward the use of biofertilizers due to all these facts, which provide nutrition through natural processes like zinc, potassium and phosphorus solubilization, nitrogen fixation, production of hormones, siderophore, various hydrolytic enzymes and protect the plant from different plant pathogens and stress conditions. They provide the nutrition in adequate amount that is sufficient for healthy crop development to fulfill the demand of the increasing population worldwide, eco-friendly and economically convenient. This review will focus on biofertilizers and their mechanisms of action, role in crop productivity and in biotic/abiotic stress tolerance.

## Introduction

The world population will reach 9 billion by 2050 in accordance with Food and Agricultural Organization; as a result, there should be an enhancement in crop yield to meet the food demand. Soil is an important source of food production in human lifespan. In the last decades, due to the increase in agricultural practices such as pesticides and chemical fertilizers it has been degraded at a universal scale and causes lower fertility due to loss in biodiversity, water retention, and disturbance in biogeochemical cycles. Soil health and plant productivity are severely influenced by numerous interactions among plant, soil, and microorganisms (Harman et al., 2020). Soil microbes cooperate with one another and also with plant roots in numerous means providing a wide variety of essential acts which are valuable for sustaining the ecological balance in soil (Kumar et al., 2021c). Plant microbial interactions are positive if they improve plant survival, nutritional status, and crop productivity and they are negative if they reduce plant growth. Soil fertility is inextricably linked to the balance of microorganisms and plants (Vishwakarma et al., 2020). The application of biofertilizers can be a probable approach to improve soil microbial status that stimulates the natural soil microbiota therefore influencing nutrient accessibility and decomposition of organic matter (Chaudhary et al., 2021). It was observed that the supply of biofertilizers in apricot modifies the microbial composition and degradation process which could be efficient in nutrient cycles in soil under field conditions (Agri et al., 2021; Baldi et al., 2021). The capability of biofertilizers to form a high-level microbial diversity in soil may outcome better crop productivity for sustainable agriculture (Agri et al., 2022). Recently, many studies reported the positive impact of beneficial soil microbes on crop productivity, but the role of consortium in agriculture is not entirely unstated. Usage of consortium has positive impact on nutrient uptake efficiency by plants, protection from pathogens, and stress conditions (Aguilar-Paredes et al., 2020). This review provided information on effective approaches such as biofertilizers which help in the restoration of agricultural soil thus improving crop health for sustainable agriculture. This can permit agriculturalists to enhance farming and reach a high standard of soil quality and subsequently lead to raised plant development.

Nutrients are required by every living creature in this world. A total of 17 essential plant nutrients are mandatory for the proper development of plants (Kumar et al., 2021a). These 17 nutrients are divided into three classes based on the amount required such as major nutrients (carbon, hydrogen, oxygen, nitrogen, phosphorus, and potassium), minor nutrients such as sulfur, calcium, and magnesium, and micronutrients (nickel, zinc, molybdenum, manganese, iron, copper, chlorine, and boron). The plant takes up oxygen, hydrogen, and carbon from air and water, but the other nutrients are taken from soils in inorganic forms (Gong et al., 2020). Biofertilizer or biological fertilizer is a material that contains living or dormant microorganisms that colonize the rhizosphere or present inside the plants and directly or indirectly promotes the growth of plants by supplying nutrition (Malusa and Vassilev, 2014; Fasusi et al., 2021). Microorganisms present in soil used as biofertilizers can mobilize the nutrient from soil and convert them into a usable form from unusable form through biological processes like nitrogen fixation, phosphorus solubilization, zinc solubilization, siderophores production, and producing plant growth-promoting substances (Bhattacharjee and Dey, 2014; Mazid and Khan, 2015). Biofertilizers are applied to seed, root, soil, or by the foliar spray to enhance the microbial activity through their multiplication which then mobilizes the nutrients to target plants which remarkably improved the soil fertility and sooner increases the crop health and production (Pandey and Singh, 2012; Ismail et al., 2013).

Biotic stress is responsible to damage plants by pathogenic organisms like bacteria, fungi, viruses, parasites, and insects and by other harmful plants. They lead to declining the crop productivity by causing diseases such as vascular wilts, leaf spots, cankers, nutrient deficiency, systematic damage, chlorosis, stunting and reduce plant vigor, ultimately causing the death of the plants (Iqbal et al., 2021). Plant protects themselves to biotic stress *via* direct mechanisms like synthesis of secondary metabolite, hormones, cell-wall-degrading enzymes, and antioxidants (Kaur et al., 2022). The indirect mechanisms include the induction of acquired systematic resistance, plant pathogen molecular patterns (PAMPs) which in turn trigger the immunity and plant resistance proteins (Yu et al., 2022). Microorganisms solubilize the phosphorus and zinc, fixing the nitrogen and other macro- and micronutrients which promote the growth of the plants under biotic stress condition by providing nutrition (Singh et al., 2022a). They also enhance the stress resistance in plants by expressing the gene of phytohormones and stress-related metabolite. Some microorganisms also produce the volatile organic compounds (VOCs) such as melatonin to protect the plant from pathogens (Moustafa-Farag et al., 2019). When pathogen attacks, the plant produces various compounds within the tissues that lead to the activation of defense mechanisms inside the plants such as induced systematic resistance, peroxidases, phenylalanine ammonia-lyase, polyphenol oxidase, and hypersensitivity (Kaur et al., 2022).

Climatic change is one of the major factors for enhancing abiotic stress on crops which results in reduced crop productivity (Liu et al., 2017a). Climatic-related abiotic stresses included drought, waterlogging, excessive heat, and soil-related abiotic stresses are fertility, heavy metals, and salinity; all these are responsible for the poor yields of crops around the whole globe (Upadhyay et al., 2019). There is less water available to plants during drought conditions, and biofertilizers have the potential to produce cytokinin, gibberellins, abscisic acid, and IAA, which cause the plant to increase its growth, root length, total surface area, and the formation of root hairs and lateral roots, which increases water absorption from water-deficient soil (Kenneth et al., 2019; Raza et al., 2019). Pollutants released from industry without any further operation if released in the environment then they cause the accumulation of heavy metals such as copper, lead, nickel, zinc, etc., which have detrimental effects on the plants and animals (Popp et al., 2013). These heavy metals are removed from the environment by micro- and macro-nutrient solubilizing and mineralizing microorganisms (Bhojiya et al., 2021). Heat stress causes cellular changes like production of reactive oxygen species, reduction in cell turgidity, reduction in water uptake, reduction in growth of plants, ultimately leading to death of plant by showing initial symptoms like leaf senescence, damages to chloroplast, wilting of plant, and chlorosis (Ahluwalia et al., 2021), whereas low temperature causes the inactivation of protein and reduces the cell membrane fluidity leading to increases in photosynthesis, imbalance of water transport (Odoh et al., 2020). All these temperature-related stresses coped up by plants after the accumulation of the hydrophilic and osmolytes protein. Huang et al. (2015) reported that due to high salt concentration there is increased toxicity to cell due to accumulations of sodium and chloride ions inside the cell which in turn disturb the photosynthetic processes, stomatal opening and closing, shrinkage of cell within pant tissue. Various studies showed that bacteria and arbuscular mycorrhizae fungi help in surviving the plants under salinity stress condition by enhancing the plant growth and development. In this review, we will discuss about the biofertilizers and its mechanism for crop production and biotic/abiotic tolerance for sustainable agriculture.

## Biofertilizers

**Figure 1:**
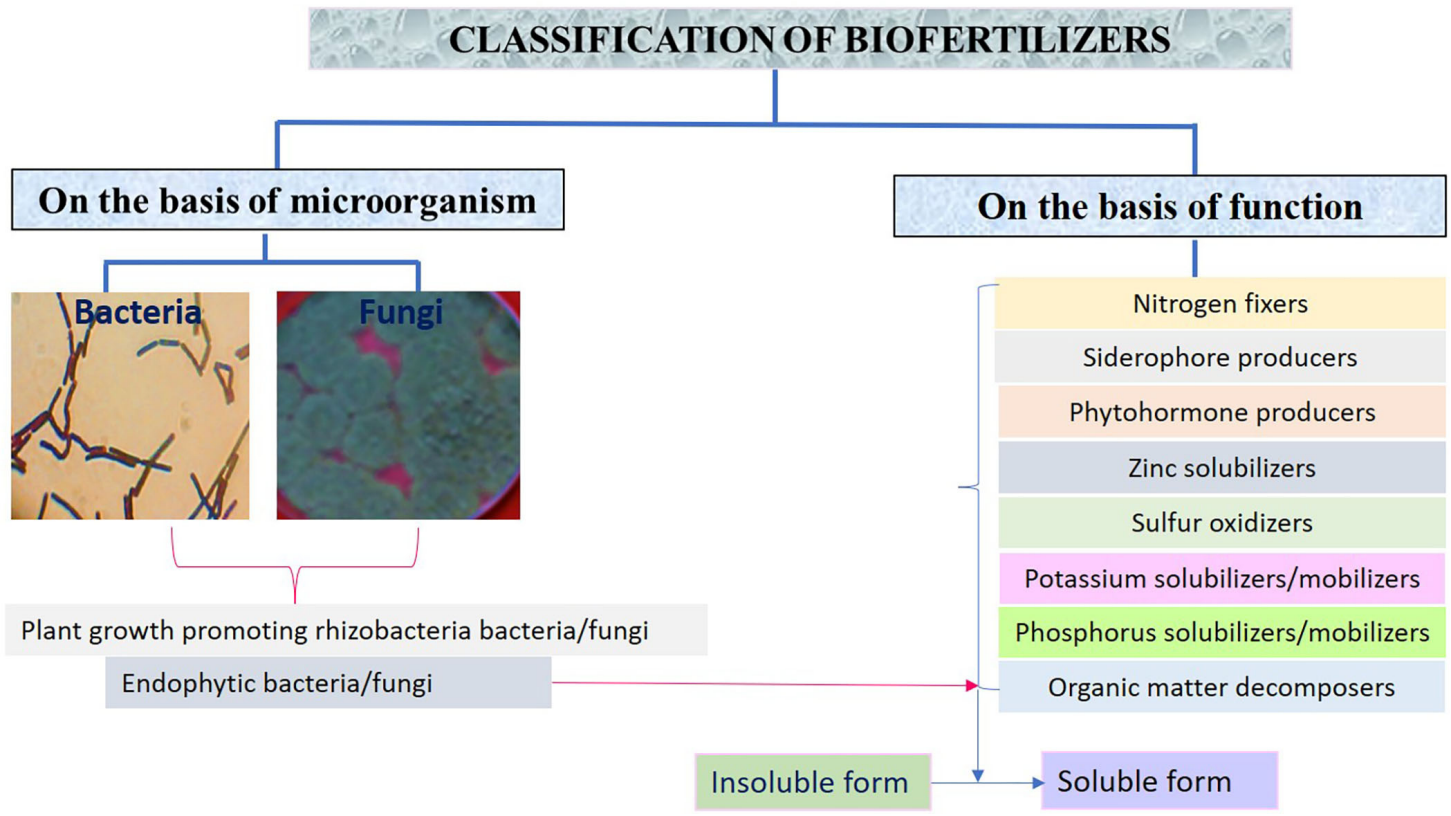
Types of biofertilizers on the basis of microorganism and functional characteristics.

In India, biofertilizer refers to the use of microorganisms to meet nutritional needs, whereas in other countries, the term microbial bioinoculant is used (Mitter et al., 2021). Biofertilizers are bio-based organic fertilizers that either could be from plant or animal sources or from living or dormant microbial cells that have the potential to improve the bioavailability and bioaccessibility of nutrient uptake in plants (Lee et al., 2018; Abbey et al., 2019). Bhardwaj et al. (2014) reported that live microbial mass is a major ingredient of biofertilizers. So biofertilizers are properly defined as “the preparations containing live microbes that help in enhancing soil fertility by fixing atmospheric nitrogen, solubilizing phosphorus or decomposing organic wastes or by elevating plant growth through the production of growth hormones with their biological activities” (Okur, 2018). Biofertilizers are generally applied in solid or dry forms, which are prepared after packing on suitable carriers such as clay minerals, rice bran, peat, lignite, wheat bran, humus, and wood charcoal. Carriers increase the shelf life and enable the easy handling of microbial inoculants (Bhattacharjee and Dey, 2014). The benefits of biofertilizers include low cost, enhanced nutrient availability, improved soil fertility, protect plants from soil-borne pathogens, sustainable agricultural production, enhanced biotic and abiotic stress tolerance, promote phytohormone production, improve soil health, causing less environmental pollution, and its continued use improves the fertility of soil considerably (Chaudhary et al., 2021, 2022a). Based on the source and raw material, global biofertilizer is marketed under two major categories like organic residue-based biofertilizer and microorganisms-based biofertilizer. Green manure, crop residues, treated sewage sludge, and farmyard manure are generally organic-based biofertilizers. While on the contrary, microorganism-based biofertilizers contain beneficial microorganisms like bacteria, fungi, and algae. Directly or indirectly, these biofertilizers mediate the performance of plant growth (Figure 1). Direct mechanisms that act upon plants directly include nitrogen fixation, phosphate solubilization, micronutrient solubilization, and the production of phytohormones (Chaudhary et al., 2021). The indirect mechanism generally protects the plant from the deleterious effect of the pathogens by releasing lytic enzymes, antibiotics, siderophores, and cyanide production (Mahmud et al., 2021).

## Types of biofertilizers and their role in crop production and soil health maintenance

Various types of biofertilizers are classified based on microorganisms such as bacteria and fungi and function of the biofertilizers as shown in Figure 1.

### Nitrogen-fixing biofertilizers

Nitrogen is the vital macro-nutrient essential by plants because it improves the growth of the shoot system, helps in reproduction, is a constituent of chlorophyll responsible for the deep green color, and also increases the size of the grains (Sandhu et al., 2021). Although the nitrogen content in the atmosphere is 78% by a mass fraction, dinitrogen contains triple bonds and is an unavailable form of nitrogen present in the air for the plants. Dinitrogen should be first converted into soluble non-toxic form ammonia by the diazotrophs through the biological process of nitrogen fixations (Abbey et al., 2019). This ammonia is then converted to the nitrite and nitrate by the ammonia-oxidizing bacteria and by nitrifying bacteria, respectively (Roy et al., 2020). The unused nitrate is converted to the atmospheric nitrogen in the deeper soil horizons through the process of denitrification which will then escape to the atmosphere as dinitrogen gas. This is the typical path of the nitrogen cycle (Mahanty et al., 2017). *Azotobacter* and *Bacillus* sp. are involved in N fixation, growth promotion of maize plants, and forest crops (Etesami et al., 2014; Azeem et al., 2022). Inoculation of *Bradyrhizobium japonicum* in soybean plants improved plant biomass, nodulation, and N fixation (Htwe et al., 2019). *Azotobacter chroococcum* improved the plant height and chlorophyll content in maize plants (Jain et al., 2021). *Bradyrhizobium* sp. showed nitrogen fixation, IAA, and siderophores production and improved the yield of mung bean (Alkurtany et al., 2018). Nitrogen-fixing microbes are considered as symbiotic, free-living, and associative nitrogen-fixing bacteria (Aasfar et al., 2021). Jing et al. (2020) reported that the application of *Pseudomonas protegens* promoted plant growth in nitrogen-deficient conditions.

### Symbiotic nitrogen-fixing microbes

In the process of symbiosis, macro-symbiont is the plant and microsymbionts are the prokaryotic bacteria. *Rhizobium* and legume symbiosis is one of the most studied mutualistic relationships between plant root nodules and nitrogen-fixing microorganisms. Mutualistic relationships are initiated when the plant began to secrete the flavonoids and iso-flavonoids in its rhizosphere, where it is recognized by *Rhizobium* (Hawkins and Oresnik, 2022). It started to do infection by differentiating root hairs, developing infection thread up to the root hair cell where infectious thread releases all its bacteria in the cytoplasmic region. Then, bacterial cell are terminally differentiated into the bacteroides, and the further development of bacteroides leads to the formation of symbiosome which is the site of nitrogen fixation (Cissoko et al., 2018; Jimenez-Jimenez et al., 2019; Suzaki et al., 2019). This atmospheric nitrogen fixation inside the nodule is carried out by the nitrogenase enzyme (Brahmaprakash and Sahu, 2012). Examples include *Rhizobium* associated with leguminous plants, *Frankia* (actinomycetes) associated with non-leguminous plants (*Alnus, Casuarina*), *Azolla* and the blue-green alga *Anabaena azollae*, and association of cyanobacteria with gymnosperms (Ghodhbane-Gtari et al., 2021). Fixation of N helps to improve the soil fertility and crop productivity. Mondal et al. (2020) reported that *Rhizobium meliloti* involved in N_2_ fixation produced chitinase enzyme and improved the yield of peanut plants. The alfalfa-Rhizobium symbiotic system can stimulate plant N fixation, increase phytohormone production, and promote plant growth (Fang et al., 2020).

### Free-living nitrogen-fixing bacteria

Mostly *Azotobacter* is studied because it is a free-living, non-symbiotic, and phototropic bacterium. *Azotobacter chroococcum* can be used as a biofertilizer because it has the potential to fix 10 mgN/g of carbon source supplied *in-vitro* (Mukherjee et al., 2022). Plant hormones such as indole acetic acids, gibberellic acids, naphthalene acetic acid, and vitamin B complex are produced by *Azotobacter*. It inhibits the root pathogens while promoting root growth, helps in mineral uptake, and improves soil fertility (Sumbul et al., 2020). Examples include *Azotobacter, Bacillus, Clostridium*, and *Azospirillum*. Application of *Bacillus* sp. significantly enhanced the growth of *Arachis hypogea* plant, protects plants from stress, and exhibits the production of ammonia and IAA (Gohil et al., 2022). *Azospirillum brasilense* reduces N fertilization, improves plant nutrition, and increases plant biomass and wheat grain yield as reported by Galindo et al. (2022).

### Associative nitrogen-fixing bacteria

The *Spirillum lipoferum* was firstly isolated by M.W. Beijerinck in 1925. *Spirillum* was found associated with the roots of the grain which were also capable of fixing nitrogen (Soumare et al., 2020). *Azospirillum* is gram-negative, non-nodulating, aerobic-associative nitrogen-fixing bacteria with plants having a C4 dicarboxylic pathway of photosynthesis, such as sugarcane, maize, sorghum, bajra, and cereals like wheat, rice, barley (Yasuda et al., 2022). They also produce cytokinin, gibberellins, and indole acetic acid, which aid in the uptake of N, P, and K and promote the growth of roots. Examples such as *Gluconobacter, Acetobacter, Herbaspirillum*, and *Azoarcus*.

### Phosphorus-solubilizing biofertilizers

Phosphorus is the second macro-nutrient that is responsible for limiting the growth of plants (Bechtaoui et al., 2021). It is an important constituent of organic and nucleic acids and is responsible for the synthesis of ATP and several amino acids. P helps in the nodulation process, amino acid synthesis, and proteins in leguminous plants (Wang et al., 2020). Soluble form of phosphorus is phosphate anion (orthophosphate), and their uptake is facilitated by rhizospheric microbes which help in plant nutrition. There are different microbes which can solubilize the remaining unavailable form of P into available form *via* organic acid production by bacteria which lowers the pH of the soil, leads to the dissolution of the phosphate compounds, and makes them available for the plant's nutrition (Mahanty et al., 2017). Examples of phosphate-solubilizing bacteria and fungi (PSB and PSF) are *Bacillus, Rhizobium, Aerobacter, Burkholderia, Aspergillus*, and *Penicillium*. Inoculation of *Alcaligenes* sp. improved plant growth parameters *via* P solubilization and IAA production (Abdallah et al., 2016). *Rhizobium leguminosarum and Pseudomonas moraviensis* enhanced the yield and growth of wheat plants and showed IAA and solubilization (Igiehon et al., 2019; Fahsi et al., 2021). Application of Arbuscular fungi can make greater availability of P in plants and protects them from stress condition as reported by Nacoon et al. (2020). *Bacillus subtilis* is also known as PSB which improved safflower growth and protects plants from salinity stress as reported by Zhang et al. (2019). NanoPhos containing phosphate-solubilizing bacteria enhanced the maize production *via* increasing the soil enzymes and bacteria population under field conditions (Chaudhary et al., 2021).

### Phosphorus-mobilizing biofertilizers

They are beneficial bacteria that effectively mobilize the soluble phosphorus and mineralization of the organic phosphorus compound, both are unavailable form of phosphorus. *Bacillus, Pseudomonas*, and *Rhizobium* are representative phosphorus-mobilizing microorganisms (PMB) (Kirui et al., 2022). Three different mechanisms have been reported for this process. First, PMB is releasing the phosphatases enzyme. Second, PMB is producing organic acids. The last one added PMB may interact symbiotically with the other fungal mycorrhiza which mobilizes the soluble phosphorus from distant places where plant roots cannot reach by absorbing soluble phosphate by hyphae (Nassal et al., 2018; Etesami et al., 2021). One of the major advantages of Arbuscular mycorrhiza is transporting both inorganic and organic forms of phosphorus to plants. Examples of arbuscular mycorrhiza fungi (AMF) include *Acaulospora* sp., *Glomus* sp*., Entrophospora*, and *Paraglomus* sp. and ectomycorrhiza include *Amanita, Laccaria*, and *Boletus* spp. Fungal endophyte (*Serendipita*) increased the K content in maize and protects plants from salinity stress (Haro and Benito, 2019).

### Potassium-solubilizing biofertilizers

Subsequently, potassium (K) is the third major constituent of the macro-nutrients required by plants. It is mainly intricate in the regulation of stomatal closing and opening, nutrient uptake, protein synthesis improving the quality of products and provides resistance against stress environment (Santosh et al., 2022). K is present in different forms in soil depending upon the type of the soil composition like water-soluble, available form, and non-available form of the K (Basak et al., 2022). K is present in immobilized forms in silicate minerals like illite, orthoclase, biotite, illite, feldspar, etc. K solubilization occurs by both bacteria and fungi, and the major mechanism for solubilization of the unavailable form of K is acidification (means release of organic acids) (Varga et al., 2020). There are mechanisms also for solubilization of the K, namely, siderophores production, exchange reaction, and complexation (Sattar et al., 2019). Examples of potassium-solubilizing bacteria include *Bacillus mucilaginous, B. edaphicus, B. circulans, Acidithiobacillus ferrooxidans, Frateuria aurantia, Herbaspirillum spp*., and *Clostridium spp.*, and potassium-solubilizing fungi include *Aspergillus spp*. and some arbuscular mycorrhiza fungi. *Bacillus cereus* showed K solubilization and improved potato plant health parameters and yield (Ali et al., 2021). Dal et al. (2020) reported that the combination of *Rhizophagus irregularis* and *A. vinelandii* improved soil enzyme activities and plant growth of wheat plants *via* P and K solubilization.

### Potassium-mobilizing biofertilizers

The potassium-mobilizing microorganisms (PMMs) effectively release the unavailable potassium through the solubilization process (Patel et al., 2021). PMM is also recognized as potassium-dissolving bacteria or potassium-solubilizing bacteria. Ghaffari et al. (2018) observed that *Frateuria* and *B. megaterium* are efficient K-mobilizing bacteria used for crop farming purposes. *Azotobacter* showed K mobilization and solubilization in wheat plant and improved growth and soil microbial activities as reported by Game et al. (2020). *Enterococcus* and *Pseudomonas aeruginosa* also showed P and K solubilization and improved the maize height, yield, and nutrient acquisition (Kumar et al., 2021b). *Bacillus aryabhattai* showed K solubilization, protects plants from stress, and improves their growth *via* the expression of K-solubilizing genes (Chen et al., 2022).

### Sulfur-solubilizing biofertilizers

Sulfur helps in chlorophyll formation, activation of a certain enzyme, amino acid formation, vitamin formation and promotes nodulation, vital for the development of all plants (Wang et al., 2019). Sulfur solubilizers are also known as sulfur-oxidizing bacteria because they are transforming the most insoluble form of sulfur that is hydrogen sulfide (H_2_S) into an available form of sulfur known as sulfate (SO4^−2^), and the reverse of this process is known assimilatory sulfate reduction which is mediated by sulfate-reducing bacteria (Wang et al., 2019). Sulfur transformation in the soil is primarily due to the microbial activity through the processes of mineralization, immobilization, oxidation, and reduction (Malik et al., 2021). Examples of aerobic sulfur-oxidizing bacteria include *Bacillus, Beggiatoa, Aquifer, Paracoccus, Sulfolobus, Thiobacillus, Thermithiobacillus, Xanthobacter;* phototropic anaerobic sulfur-oxidizing bacteria include *Allochromatium, Chlorobium, Rhodobacter, Rhodopseudomonas;* non-phototrophic obligate anaerobes include *Wolinella succinogenes;* and aerobic sulfur-oxidizing archaea include *Sulfolobales* members (Kusale et al., 2021). *Thiobacillus thiooxidans* and *Bradyrhizobium japonicum* are sulfur-oxidizing biofertilizers which showed better effect on cereal crops, medicinal plants, and forage crops (Zhang et al., 2018). *Halothiobacillus* bacteria tolerated the high salt concentration and improved crop production in saline soils (Boroujeni et al., 2021).

### Zinc-solubilizing biofertilizers

Zinc is required during protein synthesis, DNA–protein interaction, growth hormone production, seed development, production of chlorophyll and protects plants from stress conditions (Umair Hassan et al., 2020). Insoluble forms of zinc are mostly ZnO, Zn_3_(PO_4_)_2_, ZnCO_3_, and metallic Zn. The usable form of zinc by the plant is divalent cations (Ayoub et al., 2022). Zinc-solubilizing fertilizers contain the zinc solubilization bacteria which produce the organic acids to solubilize the insoluble zinc to Zn^+2^, thereby enhancing zinc uptake in plants (Nitu et al., 2020). Examples of zinc-solubilizing bacteria and fungi are *Bacillus subtilis, Pseudomonas striata, Serratia, Burkholderia cenocepacia, Aspergillus niger, A. nomius*, and *A. oryza* which improved the soil enzyme activities and availability of Zn in crop plants (Batool et al., 2021). *Leclercia adecarboxylata* solubilizes Zn and produced siderophores which enhanced the Zn uptake in the roots of cucumber plants (Kang et al., 2021). *Bacillus* spp. and *Pseudomonas taiwanensis* showed a positive impact on the growth and chlorophyll content of maize plants (Chaudhary and Sharma, 2019; Hussain et al., 2020). Inoculation of *Trichoderma longibrachiatum* and *Bacillus megaterium* improved the seed germination of soybean plants in the pot experiment (Bakhshandeh et al., 2020). The application of PSB along with fertilizers improved the growth of faba bean in sandy soils (Ding et al., 2021).

### Phytohormone-producing biofertilizers

Plant hormone or phytohormone plays a substantial role in plant development, secreted by both plants and microorganisms (Usman et al., 2022). Plant hormone production is an important feature of the beneficial microbes which is producing the indole-3-acetic acids, gibberellins, cytokinin, etc. (Eichmann et al., 2021). Auxin helped in the differentiation and division of plant cells. Cytokinin prevents the premature leaf senescence of plants (Wu et al., 2021a). Abscisic acid is also identified as hormone which is produced by plants during stress conditions. Gibberellins are involved in seed germination, shoot elongation, flowering, and fruiting (Binenbaum et al., 2018). These hormones are generally secreted by microorganisms under environmental stress conditions to protect the plants by modulating the phytohormone level inside the host plants (Lopes et al., 2021). *Bacillus thuringiensis* has the genes required for IAA production which improved the growth of tomato plants (Batista et al., 2021). *B. licheniformis* is known for the production of IAA, ABA, and gibberellin which improved the growth of grapevine and protects plants from stress conditions (Salomon et al., 2014).

### Siderophores producing biofertilizers

Iron (Fe) is a micronutrient that performs various functions like photosynthesis, respiration, chlorophyll, and many of the enzymatic reactions in plants (Gao et al., 2022). The unavailable form of iron in nature present under aerobic environment predominately is Fe^+3^ and is more probable form of insoluble oxyhydroxides and hydroxides complex. So, bacteria are producing the low-molecular weight iron-binding protein molecules called siderophores (Lurthy et al., 2020). Siderophores are water-soluble molecules that exist in two forms, namely, extracellular and intracellular. After capturing Fe^+3^ by siderophore inside bacteria, Fe^+3^ is reduced to the Fe^+2^ inside the cytoplasmic membrane which is then transported inside the cytoplasm by gating mechanisms (Gu et al., 2020). This available form of iron is given by the bacteria to the host plant for its development (Mahanty et al., 2017). Plant assimilates the iron with the help of siderophores by releasing the chelating agent *via* bacteria. Examples include *Pseudomonas fluorescens* C7*, Pseudomonas aeruginosa* RSP5, and *Pseudomonas aeruginosa* RSP8. Application of siderophore-producing *Bacillus* sp. improves the growth of groundnut (Sarwar et al., 2020). *Pseudomonas koreensis* inoculation in maize plants inhibited the growth of plant pathogens *via* the production of siderophore and antioxidant enzymes (Ghazy and El-Nahrawy, 2021).

### Organic matter decomposer biofertilizers

Soil organic matter is a mixture of living organisms consisting of bacteria, fungi, and insects, and the non-living part which includes fresh organic residues or waste, the dead and decaying matter of living organisms is generally known as humus (Lou et al., 2022). In organic matter generally, cellulose, lignin, hemicellulose, chitin, and lipids are present which are degraded by microbes such as bacteria, actinomycetes, and fungi. The organic-matter-degrading organisms break down the SOM into simpler or inorganic from which they derive energy and carbon for their growth. Examples of bacteriainclude *Bacillus subtilis* and *Pseudomonas fluorescens* and of fungi include ectomycorrhizal fungi. *Trichoderma* spp. involved in the degradation of litter at a faster rate releases antimicrobial compounds, improves the physicochemical properties of soil, and improves microbial diversity (Baldi et al., 2021). *Bacillus subtilis* and *B. hisashii* are involved in lignocellulose biodegradation by secreting the microbial enzymes as reported by Niu and Li (2022).

### Endophytic bacteria as biofertilizers

Mutualistic microorganisms that employ the whole or part of their life cycle inside the plant tissues are known as endophytes (Fadiji and Babalola, 2020). Endophytes are of interest because they improve the nutritional requirements of the non-leguminous and leguminous plants by nitrogen fixation, phosphate solubilization, or by siderophores production (Janati et al., 2021). These bacteria have the potential to suppress pathogenic effects by activating the plant defense system (Dicko et al., 2021). Examples of endophytic bacteria include *Klebsiella spp., Pseudomonas spp., Serratia spp., Bacillus spp., Burkholderia spp., Citrobacter spp*. and endophytic fungi include *Colletotrichum, Fusarium, Alternaria*, and *Aspergillus. Penicillium* and *Aspergillus* isolated from roots of *Taxus wallichiana* solubilized P and produced phosphatase and phytase enzymes (Adhikari and Pandey, 2019). Kang et al. (2014a) observed that *Bacillus megaterium* regulates the content of amino acids and carbohydrates to promote the growth of mustard plant. Endophytes isolated from rice such as *Bradyrhizobium* sp*., Paraburkholderia* sp., showed acetylene reduction properties and high sugar content contributing to high nitrogen-fixing ability. High content of sugar in different crops such as sweet potato, pineapple, and sugar has known to assist endophytic N-fixing activity among non-leguminous plants (Okamoto et al., 2021).

### Plant growth-promoting rhizobacteria

PGPR is used as biofertilizers; it represents the variation of soil bacteria that live in association with the rhizosphere, rhizoplane associated to root surface, and endophytes present inside the intercellular places (Vandana et al., 2021). PGPRs are soil bacteria which increase the growth and enhance the tolerance of plants toward stress conditions (Ghosh et al., 2019). There are diverse mechanisms shown by PGPR which support the plant growth such as N_2_ fixation, macro- and micronutrient mineralization, secretion of exopolysaccharides, phytohormone production, siderophore, hydrogen cyanide to prevent the growth of phytopathogens, antibiotics, etc. (Gouda et al., 2018; Numan et al., 2018). *Rhizobium lupini* increased alfalfa growth and enhanced nutrient uptake efficiency (Duan et al., 2022). Application of biofertilizers such as *Pseudomonas taiwanensis, Bacillus* spp., and *Pantoea agglomerans* improved the maize growth, yield, and soil health parameters (Khati et al., 2018; Chaudhary et al., 2022b). Application of *Bacillus* spp. improved the plant/soil health parameters and maize productivity as reported by Chaudhary et al. (2021). Kukreti et al. (2020) reported that *Pseudomonas taiwanensis* improved maize plant health and soil enzyme activities in the pot experiment.

## Role of biofertilizers in biotic stress management

The outbreak of plant diseases in nature necessitates sustainable agriculture with minimum use of agrochemicals. For a long time, the use of chemicals has posed a significant risk to the environment and the agricultural sector (Akanmu et al., 2021). Long-term use of pesticides, on the other hand, harms both plant/soil health and eventually leads to significant crop loss. Thus, effective and eco-friendly phytopathogen control strategies such as biofertilizers are required. The exploitation of potential biofertilizers as endophytes could be useful to improve crop plants from various bacterial and fungal diseases (Collinge et al., 2022). Biological control of plant diseases occurs *via* destruction of pathogens *via* beneficial microbes such as *Bacillus* spp*., Pseudomonas* spp*., Streptomyces, Pantoea* spp., and several fungal spp. (Köhl et al., 2019; Chaudhary et al., 2021). Such endosymbiont group of biocontrol agents being friendly, they not only colonize internal plant tissue but also protect host plant throughout its life cycle without causing any apparent damage (Lahlali et al., 2022). The use of *Bacillus* sp. for crop growth promotion and biocontrol has a long history (Zhu et al., 2021). *Bacillus thuringiensis* (Bt), a producer of endotoxins that can be used as biopesticide and a source of genes for the creation of transgenic plants that are resistant to insects, is currently the most effective biopesticide on the market (Sujayanand et al., 2021).

Biofertilizers in the form of potential biocontrol agents represent a safe alternative to harmful chemicals like fertilizers, herbicides, pesticides, and insecticides (Hernández-Fernández et al., 2021). Consequently, the use of biofertilizers is receiving special attention for the management of phytopathogens that are comprised of bacteria, fungi, virus, aphids, and nematodes (Table 1). Their ubiquitous nature and the ability to reside within plant tissues make them unique, showing multidimensional interactions within the host plant (Khare et al., 2018). The biodiversity of endophytes is hyperdiverse in almost every other plant species ranging from small non-vascular plants to large conifers like *Pinus radiata* (Liu et al., 2017b). Some of the known endophytes are *Burkholderia, Stenotrophomonas, Rhizobium, Microbacterium, and Bacillus spp*. (Kandel et al., 2017).

**Table 1:** Role of biofertilizers in biotic stress tolerance.

**Biofertilizers**	**Host plant**	**Pathogen**	**Response**	**References**
*Bacillus subtilis*	*Atractylodes macrocephala*	*Ceratobasidium* sp.	Inhibit growth of pathogen and promote plant growth	You et al., 2018
*Bacillus cereus*	*Arabidopsis*	*Botrytis cinerea*	Regulates signaling pathway such as JA and MAPK	Nie et al., 2019
*Bacillus velezensis*	*Arabidopsis*	*Myzus persicae*	Protects host plant from pathogen *via* systemic resistance response	Rashid et al., 2017
*Bacillus safensis*	*Vaccinium*	*Botrytis cinerea*	Enhanced the chitinase, hydrolytic, protease production and protects plants from pathogen	Hassan et al., 2021
*Pseudomonas aeruginosa*	*Cruciferous vegetables*	*Xanthomonas campestris*	Protects plants from pathogen *via* chitinase production	Mishra and Arora, 2012
*Gluconacetobacter diazotrophicus*	*Arabidopsis thaliana*	*Ralstonia solanacearum*	Protects from pathogen and activates defense response in plants	Rodriguez et al., 2019
*Streptomyces spp*.	*Oryzae sativa*	*Xanthomonas oryzae*	Provides immunity to plants and protect from disease *via* increasing antioxidant enzymes	Hata et al., 2021
*Paenibacillus polymyxa*	*Brassica napus*	*Verticillium spp*.	Increased production of volatile fatty acids and antibiotics	Rybakova et al., 2017
*Bacillus subtilis*	*Solanum lycopersicum*	*Fusarium oxysporum*	Increased expression of auxin-related genes and improved plant growth	Samaras et al., 2021
*Trichoderma koningii*	*Nicotiana tabacum*	*Tobacco Mosaic Virus*	Enhanced proline content and pathogen-related enzymes and inhibit the growth of pathogens	Taha et al., 2021
*Aureobasidium pullulans*	Olive trees	*Colletotrichum acutatum*	Increased production of volatile fatty acids and improves seed germination	Sdiri et al., 2022
*Trichoderma harzianum*	*Zea mays*	*Curvularia lunata*	Provides protection to plants from pathogen *via* JA signaling and platelet-activating factor	Yu et al., 2015
*Bacillus amyloliquefaciens*	*Solanum lycopersicum*	Viruses	Induced SA and JA signaling and protects plants from disease	Beris et al., 2018
*Bacillus endophyticus, Pseudomonas aeruginosa*	*Solanum lycopersicum*	*Spodoptera litura*	Increased secondary metabolite, phytohormone production and improved plant growth	Kousar et al., 2020
*Bacillus subtilis*	*Solanum lycopersicum*	*Fusarium oxysporum*	Increased plant growth and suppress the growth of pathogens	Sundaramoorthy and Balabaskar, 2013
*Bacillus sp*.	*Common bean*	*Rhizoctonia solani*	Inhibit growth of pathogens *via* production of cyanogens and lytic enzymes	Kumar et al., 2012
*Pseudomonas spp*.	*Gossypium*	*Fusarium spp*.	Inhibit pathogens *via* production of HCN and enzymes	Zain et al., 2019
*Bacillus halotolerans, Agrobacterium fabrum*	*Common bean*	*Alternaria sp*.	Improved plant growth and increased chitinase, siderophore, and IAA production	Sendi et al., 2020
*P.putida*	*Solanum tuberosum*	*Phytophthora infestans*	Increased production of HCN against pathogens	Anand et al., 2020
*Aureobasidium pullulans*	*Crops*	*Botrytis cinerea, Alternaria alternata*	Increased production of volatile organic acids and inhibit pathogen growth	Don et al., 2020

There are several enzymes which protect the plants from stress conditions such as antioxidant enzymes like peroxidase (POD), polyphenol oxidase, phenylalanine ammonia-lyase (PAL), lipoxygenase, and chitinase (Cataldo et al., 2022). Lipoxygenase enzymes have its place to non-heme iron comprising dioxygenases which contribute to stress response *via* lipid oxidation. Also, it is found to act as signals for communication with the plant host, with associated endophytes and pathogens (Singh et al., 2022b). In response to pathogen attack, endophytes boost plant immunity by priming induced systemic resistance (ISR) and systemic acquired resistance (SAR) *via* several phytohormones (Romera et al., 2019; Oukala et al., 2021). Pathogenesis-related proteins with antimicrobial properties are produced and accumulated by several endophytes symbiotically living with their host plants. Many endosymbionts have the capability to complement the inefficient antioxidative system of plants by different mechanisms (Shukla et al., 2022). In some strains, production of lipopeptides, surfactin, plipastatin, and mycosubtilin differentially activated the plant innate immune response (Kumar et al., 2021c). Production of surfactin may have an important role in the suppression of *Fusarium* infestation on germinating seeds (Eid et al., 2021). *Bacillus* strains inhibited the verticillium wilt caused due to *Verticillium dahliae* by the production of secondary metabolites such as surfactin, fengycin, and bacillibactin, as well as expressing defense-related genes such as SOD and PAL (Hasan et al., 2020). *Bacillus atrophaeus* inhibits *Meloidogyne incognita* growth by producing volatile dimethyl disulfide and antioxidant enzymes (Ayaz et al., 2021). According to Nie et al. (2019), *Bacillus cereus* inhibits the growth of *Pseudomonas syringae* by producing antioxidant enzymes. *Pseudomonas fluorescent* controls the iron uptake genes and protects plants from phytopathogens as reported by Desrut et al. (2020). *Acrophialophora jodhpurensis* defends tomato plants from *Rhizoctonia solani* which causes crown root disease *via* the production of peroxidase enzyme, chitinase, and phenylalanine (Daroodi and Taheri, 2021). Plants are protected from *Botrytis cinerea* by *Trichoderma atroviride via* the production of glutamate and glyoxylate aminotransferase (González-López et al., 2021).

Secondary metabolites play the foremost role in defense mechanism toward pathogens, pests, and herbivores. Many plants microbiome especially endosymbionts regulate defense mechanisms through secreting various metabolites (Divekar et al., 2022). Secondary plant metabolites belonging to the family of steroids, alkaloids, phenolics, flavonoids, and terpenoids function in innate immunity and defense response signaling (Pang et al., 2021). Volatile compounds from endophytes modulate plant microbiome and possess antimicrobial properties. A variety of fungi, including *Ascomycetes* and *Deuteromycetes*, are inhibited by a mixture of VOCs produced by the fungal endophyte *Phomopsis* sp. (Hummadi et al., 2022). Three VOCs, including caryophyllene, 2-methoxy-4-vinylphenol, and 3,4-dimethoxystyrol, produced by endophytic fungi *Sarocladium bravhiariae* HND5 have been found to have antifungal activity against *Fusarium oxysporum* (Yang et al., 2021). Alkaloid produced by *Epichloe* sp. in a variety of grass species is one of the well-known secondary metabolites produced by endophytic fungi. Hennessy et al. (2022) reported *Epichloe festucae* colonized agricultural forage grasses and offered the plant defense against herbivorous insects. *Streptomyces hydrogenans* metabolites can be used as safe biocontrol agents against *Meloidogyne incognita* and plant growth promoters for *Solanum lycopersicum* (Sharma et al., 2020). *Bacillus velezensis* is a potential pesticide due to its strong biocontrol activity and ability to strengthen host defense against *Magnaporthe oryzae* fungi, which cause rice blast disease in plants (Chen et al., 2021). An isolate of *Trichoderma asperellum* increased the resistance in tomato seedling to the disease *A. alternata* leaf spot (Yu et al., 2021). *Trichoderma asperellum* also produces mycolytic enzymes such as chitinase and 1,3, glucanase which may be capable of destroying phytopathogens cell walls (Win et al., 2021). *Trichoderma* spp. also have biocontrol potential against *V. dahliae*, which causes olive tree wilting, and inhibit the pathogenic fungus mycelial growth (Reghmit et al., 2021). *Trichoderma* sp. has been shown successfully to suppress *Sclerospora graminicola*, the cause of pearl millet downy mildew disease, and develop systemic resistance (Nandini et al., 2021).

Some endophytes can also regulate stress management through SAR mediated by salicylic acid. SAR offers long-lasting stress management and broad-spectrum effectiveness against a variety of pathogens (Xia et al., 2022). It frequently involves the accumulation of chitinase and pathogenesis-related proteins (PR). In a study by Samain et al. (2019), *Paenibacillus* strain (PB2) used to control *Mycosphaerella graminicola* induced pathogenesis-related proteins (PR1) which is considered as marker of SAR. Application of *Bacillus aryabhattai* activated a durable defense response against pathogens facilitated through salicylic acid/ethylene pathways (Portieles et al., 2021). *Trichoderma harzianum* helps to improve plant immediate resistance against *Nezara viridula* feeding invasion *via* enhancing JA marker gene transcript levels (Alınç et al., 2021). *Bacillus subtilis* and *Pseudomonas fluorescens* mediated systemic alleviated the biotic stress in *Solanum lycopersicum* against *Sclerotium rolfsii*. Heat-killed endophytic strain *B. aryabhattai* (HKEB) induced defense-related genes protein (PR1) and phytoalexin-deficient 3 in *A. thaliana*. PR1 gene expression was found to be 20-fold higher in treated plants than control, and other genes found in the study were associated with jasmonic and salicylic acid pathways (Portieles et al., 2021). Endophytes exhibit different gene upregulation and a distinct signaling pathway in response to distinct colonization strategies (Morelli et al., 2020). *Trichoderma* spp. demonstrated antagonistic activity against phytopathogens such as B. *cinerea, Fusarium solani*, and *Rhizoctonia solani* used as biocontrol agent in greenhouse experiment (Sánchez-Montesinos et al., 2021).

## Role of biofertilizers in abiotic stress management

Climate change is one of the major reasons for the increasing abiotic stresses on the crops, which results in reducing the world's agriculture productivity. Abiotic stresses like drought, salinity, waterlogging, and excessive heat are responsible for the poor yield of crops (He et al., 2018). In recent years, the abiotic stress has increased so fast, because of the fluctuation of climates or climate change, and it has caused an unusual rise in the weather conditions and incidents, which is responsible for the substantial losses of crops around the globe. These abiotic stresses induce several physiological, biochemical, and morphological changes in plant that finally affect the economic yield of crop plants, and it was reported that the yield loss from abiotic stress is about 51–82%, which if continues will affect the goal of sustainable food production (dos Santos et al., 2022).

**Figure 2:**
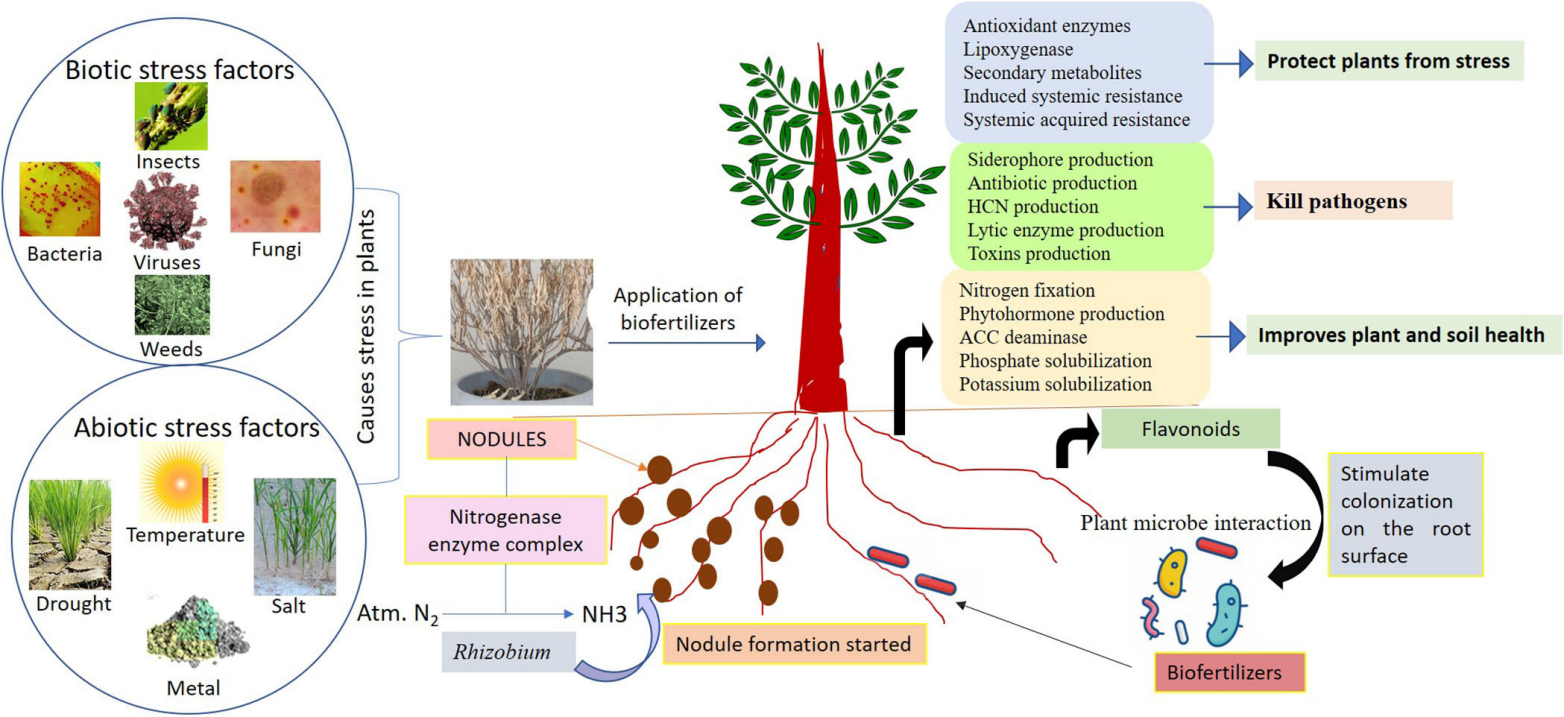
Role of biofertilizers for maintenance of crop productivity and soil health.

The use of beneficial microbes such as endophytes capable of producing growth hormones, like IAA, ACC deaminase, augmented the K uptake in plant tissues but decreased the ethylene level which helps in tolerance of stress in diverse plants (Figure 2). Biofertilizers as endophytes are found to have diverse associations with its host plant such as symbiotic, parasitic, and mutualistic and colonize plant tissues without causing any disease, thus benefiting for plants (Chaudhary et al., 2022c). Endophytes may benefit from mutualistic associations as they obtain nutrients from the hosts, and they spread by host seed transmission. They are also able to enhance the nutrients uptake like nitrogen, magnesium, zinc, and phosphorus from soil and provide to the host plant for better growth and survival (Bamisile et al., 2018). It is well-identified that plant biofertilizers play a significant role in supporting the growth of crops under different abiotic stresses (Table 2). Actinobacteria are well-known for plant growth *via* metabolite production and antibiotics under stress conditions (Yadav et al., 2018). Abd El-Daim et al. (2014) observed that the application of *Pseudomonas* sp. improved plant growth under heat stress *via* HSPs and ROS reduction. *Paenibacillus* sp. improved *Phaseolus vulgaris* growth *via* facilitating the siderophore, IAA and HCN production under salinity stress conditions (Gupta and Pandey, 2019). *Trichoderma harzianum* inoculation in rice plants improved root growth and protects from drought stress as reported by Shukla et al. (2012). Mukhtar et al. (2020a) reported that *Bacillus cereus* enhanced the production of ACC deaminase and exopolysaccharide which protects *Solanum lycopersicum* plants from heat stress.

**Table 2:** Role of biofertilizers in abiotic stress tolerance.

**Biofertilizers**	**Host plant**	**Stress**	**Response**	**References**
*Bacillus aryabhattai*	*Oryza sativa*	Salinity, heavy metals	Improved salt tolerance ability *via* exopolysaccharide production	Sultana et al., 2020
*Bacillus amyloliquefaciens*	*Arabidopsis*	Salt	Improved plant growth *via* regulation of JA pathway and antioxidant enzymes	Liu et al., 2020
*Bacillus licheniformis*	*Chrysanthemum*	Salt	Improved salt tolerance ability in stressed plants *via* production of antioxidant enzymes and Fe attainment	Zhou et al., 2017
*Bacillus HL3RS14*	*Zea mays*	Salt	Increased weight of roots and shoots *via* production of IAA, proline, and glycine betaine	Mukhtar et al., 2020b
*Bacillus subtilis, Pseudomonas sp*.	*Solanum melongena*	Salt	Increase chlorophyll content and protects plants from stress	Mokabel et al., 2022
*Bacillus sp*.	*Pisum sativum*	Salt	Improved plant growth and photosynthesis *via* antioxidant enzyme and AAC and siderophore production	Gupta et al., 2021
*Gluconacetobacter diazotrophicus*	*Zea mays*	Drought and nitrogen	Increase plant biomass and chlorophyll content	Tufail et al., 2021
*Pseudomonas pseudoalcaligenes*	*Glycine max*	Salt	Improved plant health parameters *via* production antioxidant enzyme, proline contents in shoots and roots	Yasmin et al., 2020
*Alternaria alternata*	*Triticum aestivum*	Drought	Improved photosynthesis *via* increasing antioxidant enzymes	Qiang et al., 2019
*Aspergillus flavus*	*Glycine max*	Salt	Increased antioxidant enzyme activity and chlorophyll content	Asaf et al., 2018
*Aspergillus violaceofucus*	*Helianthus annuus*	Heat	Improved plant height, biomass, and chlorophyll content	Ismail et al., 2020
*Funneliformis mosseae*	*Trifoliate orange*	Drought	Improved phenols, terpenoids and soil protein and enzyme activities	Cheng et al., 2021
*Glomus lomus*	*Date palm*	Salt	Improved shoot weight and growth	Meddich et al., 2018
*Piriformospora indica*	*Triticum aestivum*	Nutrient	Improved Zn uptake and root and shoot biomass	Abadi et al., 2021
*Piriformospora indica*	*Arabidopsis*	Cold	Increased proline content and cold stress tolerance genes	Jiang et al., 2020
*Trichoderma atroviridae*	*Arabidopsis*	Cold	Improved auxin production and cold-related gene expression	González-Pérez et al., 2018
*Rhizophagus intraradices*	C3 plants	Salt	Improved chlorophyll content in plants	Chandrasekaran et al., 2019
*AMF* and *Bradyrhizobium japonicum*	*Glycine max*	Drought	Increased yield and protects from stress *via* upregulation of CAT and POD activity	Sheteiwy et al., 2021
*AMF* and *Rhizobium* spp. *Serratia marcescens*	*Glycine max Lactuca sativa*	Drought Salinity	Improved plant health and microbial diversity in soil Triggered CAT, proline, and IAA production	Igiehon et al., 2021 Fortt et al., 2022

### Drought stress

Drought stress is one of the main abiotic stresses which causes water scarcity to meet the plant necessity and causes economic fatalities in agriculture production. The normal progress of plants is hindered due to decrease in water shortage in their cells. Drought stress decreased the rate of photosynthesis, germination in plants, and loss in crop productivity (Lata et al., 2018). Inoculation of beneficial biofertilizers (rhizospheric and endophytic microbes) improved plant growth and development *via* different direct/indirect mechanisms under stress situations. Stress can be overcome *via* using biofertilizers which produced growth hormones such as IAA and cytokinins and improved plant development (Fasusi et al., 2021). Inoculation of *Pseudomonas putida* boosted the flavonoids, salicylic, and abscisic acid production which protects soybean plants from drought stress (Kang et al., 2014b). Inoculation of *Pseudomonas* spp. protects maize plants and improved biomass and sugar content in treated plants from drought stress *via* upregulation of dehydrin proteins and proline content (Sandhya et al., 2010). Khan et al. (2018) found that *Bacillus thuringiensis* improved chickpea growth under drought conditions *via* production of volatile organic compounds. Application of *Microbacterium* sp. improved maize plant growth, root length, photosynthetic rate, and yield under drought stress (Romera et al., 2019). Usage of *Phoma* improved the drought tolerance in *Pinus tabulaeformis* plants and increased seedling growth by improving the mechanism of water uptake, proline, and SOD (Zhou et al., 2021). Sheteiwy et al. (2021) reported that *Bradyrhizobium japonicum* and AMF improved the yield of soybean bacterial count and enzyme activities of soil *via* improving the nutrient accessibility in soil under drought stress. AMF and *Rhizobium* inoculation improved the *Glycyrrhiza* plant growth and phosphorus content in roots in drought stress (Hao et al., 2019). Combined inoculation of arbuscular fungi and bioinoculants improved plant biomass and chlorophyll content in date palm (*Phoenix dactylifera*) under water-deficit conditions *via* enhanced antioxidant enzyme activities, soluble sugars, and proteins (Anli et al., 2020). Inoculation of *Glomus mosseae* and *Bacillus amyloliquefaciens* in *Phaseolus vulgaris* significantly improved the photosynthetic rate and yield under water stress conditions (Salem and Al-Amri, 2021).

### Salinity stress

Accumulation of salt in agricultural soil will have a negative impact on plants including its physiological, morphological, and molecular aspects. This affects plants *via* creating osmotic stress, ion toxicity and reducing the photosynthesis, CO_2_ fixation, and transpiration rate in plants. Availability of nutrients and microbial diversity are also affected due to the salinity stress (Luo et al., 2021). Usage of bioinoculants is enormously supportive in countering the lethal properties of soil salinity *via* improving the soil physicochemical properties and thus improved crop production (Jiménez-Mejía et al., 2022). Interaction between microbes and plants can overcome stress problem. Gond et al. (2015) reported that inoculation of *Pantoea agglomerans* in tropical corn under salt stress (0–100 mM) improves tolerance and growth of plants due to the upregulation of aquaporins. *Bacillus megaterium* also regulates the aquaporin genes during salt stress in maize plants and improved root growth and leaf water content (Marulanda et al., 2010). Waqas et al. (2012) reported that *Penicillium* and *Phoma glomerata* improved the rice plant growth under salinity stress *via* increased production of CAT, POD, and IAA. Checchio et al. (2021) observed that *Azospirillum brasilense* improved resistance in corn plants *via* enhancing the production of antioxidant enzymes and glycine betaine. Application of *Pseudomonas* sp. improves *Arabidopsis thaliana* germination and growth *via* upregulation of lipoxygenase genes which are involved in tolerance mechanism *via* jasmonic pathway (Chu et al., 2019). The *Arthrobacter nitroguajacolicus* improved wheat growth under salt stress *via* upregulation of IAA, ACC, flavonoid, stilbenoid, terpenoids, and cytochrome P450 genes (Safdarian et al., 2019). Inoculation of *Planococcus rifietoensis* protects Cicer arietinum plants from salt stress (200 mM) *via* EPS and biofilm production (Qurashi and Sabri, 2012). Gupta and Pandey (2019) observed that inoculation of *Paenibacillus* sp. protects and improved *Phaseolus vulgaris* plant growth under salinity stress *via* the production of IAA and ACC deaminase. Meena et al. (2020) reported that *Nocardioides* sp. improved seedling growth of *Triticum aestivum* under salt stress (0–100 mM) *via* increasing the CAT and POD genes. Inoculation of *Penicillium* and *Ampelomyces* spp. improved drought and salinity stress tolerance in tomato plants *via* the production of osmolytes, stress-responsive genes, and antioxidant enzymes (Morsy et al., 2020). Inoculation of *Piriformospora indica* highly enhanced plant development and attenuated NaCl-induced lipid peroxidation which helps to build tolerance during salinity stress (Ghaffari et al., 2018). Studies show that the inoculation of *Trichoderma longibrachiatum* T6 in wheat increased the levels of antioxidant enzymes (SOD, POD, and CAT) which helped to improve the stress tolerance in plants during salt stress (Zhang et al., 2016). *Agrobacterium* and *Raoultella* showed production of IAA, HCN, and ACC under salt stress and improved growth of *Tetragonia tetragonioides* plants (Egamberdieva et al., 2022). Fortt et al. (2022) reported that the application of PGPR improved the growth of lettuce under salt stress *via* the production of IAA and antioxidant enzymes which provide protection to plants.

### Temperature stress

Global warming is a serious risk to all living creatures and is becoming a worldwide concern. Temperature stress such as heat and cold greatly limits the growth and development of plants (Yadav et al., 2018). Heat stress causes modification in homeostasis, degradation of proteins, which have lethal effects on physiology of plants as it delays the seed germination, damages to seeds and affects agricultural production (Imran et al., 2021). Cold stress causes dehydration due to ice formation which is responsible for protein denaturation. It also causes plant leaves lesions, yellowing of leaves, and rotting. It also affects the seed germination and yield of crops (Wu et al., 2021b). The application of several microbes alleviated the damaging effects of heat stress in various plants such as wheat, tomato, and sorghum *via* producing phytohormones, biofilm formation, and enhancing heat shock proteins (Issa et al., 2018; Sarkar et al., 2018). *Bacillus cereus* inoculation in tomato plants increased the production of HSPs, IAA, essential amino acids, and organic acids and protects plants from stress conditions (Khan et al., 2020). Inoculation of *Azospirillum* and *B. amyloliquefaciens* improved the heat tolerance *via* reducing oxidative damage in wheat seedling (Abd El-Daim et al., 2014). Duc et al. (2018) reported that *Glomus* sp. tolerates heat stress and protects tomato plants *via* scavenging ROS generation. *Bacillus velezensis* improved wheat plant survival under cold stress *via* increase in cold stress-related proteins as reported by Abd El-Daim et al. (2019). Zulfikar et al. (2011) reported that *Pseudomonas putida* also improved the growth of wheat plants under heat stress *via* enhanced production of proline, sugars, and antioxidant enzymes. *R. irregularis* and *F. mosseae* increased plant height, transpiration rate in maize, and nutrient composition in roots of *triticum aestivum* during heat stress (Cabral et al., 2016). *Paraburkholderia phytofirmans* having ACC deaminase-producing efficiency helps in normal growth of tomato plants under heat stress as reported by Esmaeel et al. (2018). Bacterial inoculants such as *Rhodococcus* and *Burkholderia* protect the medicinal plant *Atractylodes lancea* from heat stress and improved their growth *via* enriched root-associated microbes (Wang et al., 2022a).

### Heavy metal stress

Extreme usage of inorganic chemical fertilizers in agriculture system causes the accumulation of toxic metals such as nickel, manganese, cadmium, iron, and zinc in soil (Ghori et al., 2019). These metals are beneficial for plants at low level, but if their concentration increases cause stress *via* decrease in plant growth due to the decrease in photosynthesis, deprived nutrients, membrane integrity, and enzyme activities. It causes oxidative stress *via* ROS and H_2_O_2_ generation and reduces plant growth and crop productivity (Ahmad et al., 2019; Gong et al., 2020). ROS generation occurs both under favorable and unfavorable circumstances, and it has a negative impact on vital macromolecules (Köhl et al., 2019). *Rhizobium* inoculation at nickel-contaminated site improves the chlorophyll content and increased lentil plant growth (Wani and Khan, 2013). *Bradyrhizobium* increased IAA production and siderophore production and improved the shoot weight of *Lolium multiflorum* at cadmium-contaminated site (Guo and Chi, 2014). *Candida parapsilosis* and *B. cereus* protect *Trifolium repens* plants from heavy metal stress conditions as reported by Azcón et al. (2010). Toxicity of arsenic in *Brassica juncea* is reduced by *Staphylococcus arlettae via* enhanced production of dehydrogenase and phosphatase enzyme in soil (Srivastava et al., 2013). Inoculation of *Talaromyces pinophilus in Triticum aestivum* plants stimulates plant growth *via* the production of gibberellic acid under heavy metal stress (El-Shahir et al., 2021). Paredes-Páliz et al. (2018) reported that inoculation of metal-resistant bacteria such as *B. aryabhattai* and *Pantoea agglomerans* brings production of phenylalanine ammonia-lyase enzyme and SOD which protects plants from metal stress. The addition of bioinoculants like *P. aeruginosa* and *Burkholderia gladioli* reduced Cd toxicity in *Solanum lycopersicum* by producing phenols, organic acids, and osmoprotectants (Khanna et al., 2019). Application of *Serratia marcescens* and *E. bugandensis* improved spinach (*Ipomoea aquatica*) growth *via* the production of polyamine under Pb and Cd toxicity (Wang et al., 2022b). *Citrobacter* and *Enterobacter cloacae* mitigate the Cd and Pb toxicity, improve the wheat plant health parameters, and protect from stress *via* the generation of antioxidant enzymes (Ajmal et al., 2022). Oubohssaine et al. (2022) reported that *Pseudarthrobacter oxydans* improved *Sulla spinosissima* growth and can be used as biofertilizer at heavy metal-contaminated sites. Cadmium tolerance bacteria such as *Curtobacterium ocenosedimentum* having P-solubilizing, IAA, and siderophore-producing possessions improved chili growth and increased shoot/root length (Patel et al., 2022). Inoculation of *Pseudoarthrobacter* and *Vibrio neocaledonicus* improved the *Salicornia ramosissima* growth at As- and Cu-polluted sites (Mesa-Marín et al., 2020). *Rhizobium* inoculation can promote soil nutrient cycling by increasing enzyme activity in metal-contaminated soil, thereby providing more N and P for microbial activity and growth of plants (Ma et al., 2021; Duan et al., 2022). Heavy metal toxicity is a growing problem in the world; therefore, finding appropriate microbes proficient to depollution of the metals can benefit to improve the crop efficiency. Application of biofertilizers for sustainable food crop production and boosting various stress tolerance of plants are gaining popularity. Still, further studies are crucial to unravel the potential role of biofertilizers in responding to the impact of different stresses at molecular level.

## Conclusion

Agriculture systems have to face the task of food production, stress management, and dependency on agrochemicals. The presence of pest and pathogen in crops causes decrease in crop yield and heavy crop losses every year. The occurrence of abiotic stresses due to the change in climatic conditions leads to difficult challenge to crop production worldwide. Different effective approaches should be employed to reduce crop output loss and control diseases. Hence, the necessity to implement the eco-friendly approaches such as biofertilizers is of great importance for sustainable agriculture. The application of biofertilizers not only improves plant heath parameters but also enhances the crop productivity, soil health and protects from stress environment. More research has been focused on physiological and molecular aspects under different conditions with different crops using biofertilizers under field conditions.

## Author contributions

PC: conceptualization and wrote the manuscript. SS, AC, AS, and GK: editing the manuscript. All authors contributed to the article and approved the submitted version.

## Conflict of interest

The authors declare that the research was conducted in the absence of any commercial or financial relationships that could be construed as a potential conflict of interest.

## Publisher's note

All claims expressed in this article are solely those of the authors and do not necessarily represent those of their affiliated organizations, or those of the publisher, the editors and the reviewers. Any product that may be evaluated in this article, or claim that may be made by its manufacturer, is not guaranteed or endorsed by the publisher.

## References

[B1] Aasfar A. Bargaz A. Yaakoubi K. Hilali A. Bennis I. Zeroual Y. Kadmiri M. I. (2021). Nitrogen fixing *Azotobacter* species as potential soil biological enhancers for crop nutrition and yield stability. Front. Microbiol. 12, 628379. 10.3389/fmicb.2021.628379 33717018 PMC7947814

[B2] Abadi V. A. J. M. Sepehri M. Khatabi B. Rezaei M. (2021). Alleviation of zinc deficiency in wheat inoculated with root endophytic fungus *Piriformospora indica* and *Rhizobacterium, Pseudomonas putida*. Rhizosphere 17:100311. 10.1016/j.rhisph.2021.100311

[B3] Abbey L. Abbey J. Leke-Aladekoba A. Iheshiulo E. M. A. Ijenyo M. (2019). Biopesticides and biofertilizers: types, production, benefits, and utilization. Byprod. Agri. Fisher. 2019, 479–500. 10.1002/9781119383956.ch20

[B4] Abd El-Daim I. Bejai S. Meijer J. (2014). Improved heat stress tolerance of wheat seedlings by bacterial seed treatment. Plant Soil. 379, 337–350. 10.1007/s11104-014-2063-3

[B5] Abd El-Daim I. A. Bejai S. Meijer J. (2019). *Bacillus velezensis* 5113 induced metabolic and molecular reprogramming during abiotic stress tolerance in wheat. Sci. Rep. 9:16282. 10.1038/s41598-019-52567-x 31704956 PMC6841942

[B6] Abdallah R. A. B. Trabelsi B. M. Nefzi A. Khiareddine H. J. Remadi M. D. (2016). Isolation of endophytic bacteria from *Withania somnifera* and assessment of their ability to suppress *Fusarium* wilt disease in tomato and to promote plant growth. J. Plant Pathol. Microbiol. 7:352. 10.4172/2157-7471.1000352

[B7] Adhikari P. Pandey A. (2019). Phosphate solubilization potential of endophytic fungi isolated from *Taxus wallichiana* Zucc. roots. Rhizosphere 9, 2–9. 10.1016/j.rhisph.2018.11.00232738763

[B8] Agri U. Chaudhary P. Sharma A. (2021). In vitro compatibility evaluation of agriusable nanochitosan on beneficial plant growth-promoting rhizobacteria and maize plant. Natl. Acad. Sci. Lett. 44, 555–559. 10.1007/s40009-021-01047-w

[B9] Agri U. Chaudhary P. Sharma A. Kukreti B. (2022). Physiological response of maize plants and its rhizospheric microbiome under the influence of potential bioinoculants and nanochitosan. Plant Soil 474, 451–468. 10.1007/s11104-022-05351-2

[B10] Aguilar-Paredes A. Valdés G. Nuti M. (2020). Agronomy ecosystem functions of microbial consortia in sustainable agriculture. Agronomy. 10. 10.3390/agronomy10121902

[B11] Ahluwalia O. Singh P. C. Bhatia R. (2021). A review on drought stress in plants: Implications, mitigation and the role of plant growth promoting rhizobacteria. Res. Environ. Sustain. 2021:100032. 10.1016/j.resenv.2021.100032

[B12] Ahmad P. Tripathi D. K. Deshmukh R. Pratap S. V. Corpas F. J. (2019). Revisiting the role of ROS and RNS in plants under changing environment. Environ. Experi. Botany 161, 1–398. 10.1016/j.envexpbot.2019.02.017

[B13] Ajmal A. W. Yasmin H. Hassan M. N. Khan N. Jan B. L. Mumtaz S. (2022). Heavy metal-resistant plant growth-promoting *Citrobacter werkmanii* strain WWN1 and *Enterobacter cloacae* strain JWM6 enhance wheat (*Triticum aestivum* L.) growth by modulating physiological attributes and some key antioxidants under multi-metal stress. Front. Microbiol. 6, 815704. 10.3389/fmicb.2022.815704 35602039 PMC9120770

[B14] Akanmu A. O. Babalola O. O. Venturi V. Ayilara M. S. Adeleke B. S. Amoo A. E.. (2021). Plant disease management: leveraging on the plant-microbe-soil interface in the biorational use of organic amendments. Front. Plant Sci. 12, 700507. 10.3389/fpls.2021.700507 34394153 PMC8360880

[B15] Ali A. A. Awad M. Y. M. Hegab S. A. Abd El Gawad A. M. Eissa M. A. (2021). Effect of potassium solubilizing bacteria (*Bacillus cereus*) on growth and yield of potato. J. Plant. Nutr. 44, 411–420. 10.1080/01904167.2020.1822399

[B16] Alınç T. Cusumano A. Peri E. Torta L. Colazza S. (2021). *Trichoderma harzianum* strain T22 modulates direct defense of tomato plants in response to *Nezara viridula* feeding activity. J Chem Ecol. 47, 455–462. 10.1007/s10886-021-01260-3 33713251 PMC8116274

[B17] Alkurtany A. Ali S. Mahdi W. (2018). The efficiency of prepared biofertilizer from local isolate of *Bradyrhizobium* sp. on growth and yield of mungbean plant. Iraqi J. Agric. Sci. 49, 722–730.

[B18] Anand A. Chinchilla D. Tan C. Mène-Saffrané L. L'Haridon F. Weisskopf L. (2020). Contribution of hydrogen cyanide to the antagonistic activity of *Pseudomonas* strains against *Phytophthora infestans*. Microorganisms 8:1144. 10.3390/microorganisms8081144 32731625 PMC7464445

[B19] Anli M. Baslam M. Tahiri A. Raklami A. Symanczik S. Boutasknit A.. (2020). Biofertilizers as strategies to improve photosynthetic apparatus, growth, and drought stress tolerance in the date palm. Front. Plant Sci. 11, 516818. 10.3389/fpls.2020.516818 33193464 PMC7649861

[B20] Asaf S. Hamayun M. Khan A. L. Waqas M. Khan M. A. Jan R.. (2018). Salt tolerance of *Glycine max*. L induced by endophytic fungus *Aspergillus flavus* CSH1, via regulating its endogenous hormones and antioxidative system. Plant Physiol. Biochem. 128, 13–23. 10.1016/j.plaphy.2018.05.007 29751251

[B21] Ayaz M. Ali Q. Farzand A. Khan A. Ling H. Gao X. (2021). Nematicidal volatiles from *Bacillus atrophaeus* GBSC56 promote growth and stimulate induced systemic resistance in tomato against *Meloidogyne incognita*. Int. J. Mol. Sci. 22:5049. 10.3390/ijms22095049 34068779 PMC8126219

[B22] Ayoub I. Kumar V. Abolhassani R. Sehgal R. Sharma V. Sehgal R.. (2022). Advances in ZnO: Manipulation of defects for enhancing their technological potentials. Nanotechnol. Rev. 11, 575–619. 10.1515/ntrev-2022-0035

[B23] Azcón R. Perálvarez M. C. Roldán A. Barea J. (2010). Arbuscular mycorrhizal fungi, *Bacillus cereus*, and *Candida parapsilosis* from a multicontaminated soil alleviate metal toxicity in plants. Microb. Ecol. 59, 668–677. 10.1007/s00248-009-9618-5 20013261

[B24] Azeem M. Haider M. Z. Javed S. Saleem M. H. Alatawi A. (2022). Drought stress amelioration in maize (*Zea mays* L.) by inoculation of *Bacillus* spp. Strains under sterile soil conditions. Agriculture 12:50. 10.3390/agriculture12010050

[B25] Bakhshandeh E. Gholamhosseini M. Yaghoubian Y. Pirdashti H. (2020). Plant growth promoting microorganisms can improve germination, seedling growth and potassium uptake of soybean under drought and salt stress. Plant Growth Regul. 90, 123–136. 10.1007/s10725-019-00556-5

[B26] Baldi E. Gioacchini P. Montecchio D. Mocali S. Antonielli L. Masoero G.. (2021). Effect of biofertilizers application on soil biodiversity and litter degradation in a commercial apricot orchard. Agronomy 11:1116. 10.3390/agronomy11061116

[B27] Bamisile B. S. Dash C. K. Akutse K. S. Keppanan R. Wang L. (2018). Fungal endophytes: beyond herbivore management. Front. Microbiol. 9, 544. 10.3389/fmicb.2018.00544 29628919 PMC5876286

[B28] Basak B. B. Maity A. Ray P. Biswas D. R. Roy S. (2022). Potassium supply in agriculture through biological potassium fertilizer: a promising and sustainable option for developing countries. Arch. Agronomy Soil Sci. 68, 101–114. 10.1080/03650340.2020.1821191

[B29] Batista B. D. Dourado M. N. Figueredo E. F. (2021). The auxin-producing *Bacillus thuringiensis* RZ2MS9 promotes the growth and modifies the root architecture of tomato (*Solanum lycopersicum* cv. Micro-Tom). Arch. Microbiol. 203, 3869–3882. 10.1007/s00203-021-02361-z 34013419

[B30] Batool S. Asghar H. N. Shehzad M. A. Yasin S. Sohaib M. Nawaz F.. (2021). Zinc-solubilizing bacteria-mediated enzymatic and physiological regulations confer zinc biofortification in chickpea (*Cicer arietinum* L.). J. Soil Sci. Plant Nutr. 21, 2456–2471. 10.1007/s42729-021-00537-6

[B31] Bechtaoui N. Rabiu M. K. Raklami A. Oufdou K. Hafidi M. Jemo M. (2021). Phosphate-dependent regulation of growth and stresses management in plants. Front. Plant Sci. 12, 679916. 10.3389/fpls.2021.679916 34777404 PMC8581177

[B32] Beris D. Theologidis I. Skandalis N. (2018). *Bacillus amyloliquefaciens* strain MBI600 induces salicylic acid dependent resistance in tomato plants against Tomato spotted wilt virus and Potato virus Y. Sci. Rep. 8:10320. 10.1038/s41598-018-28677-3 29985434 PMC6037670

[B33] Bhardwaj D. Ansari M. W. Sahoo R. K. Tuteja N. (2014). Biofertilizers function as key player in sustainable agriculture by improving soil fertility, plant tolerance and crop productivity. Microb. Cell Fact. 13, 1–10. 10.1186/1475-2859-13-66 24885352 PMC4022417

[B34] Bhattacharjee R. Dey U. (2014). Biofertilizer, a way towards organic agriculture: A review. Afr. J. Microbiol. Res. 8, 2332–2343. 10.5897/AJMR2013.637434480627

[B35] Bhojiya A. A. Joshi H. Upadhyay S. K. Srivastava A. K. Pathak V. V. Pandey V. C.. (2021). Screening and optimization of zinc removal potential in *Pseudomonas aeruginosa-*HMR1 and its plant growth-promoting attributes. Bull. Environ. Contamination Toxicol. 2021, 1–10. 10.1007/s00128-021-03232-5 33860803

[B36] Binenbaum J. Weinstain R. Shani E. (2018). Gibberellin localization and transport in plants. Trends Plant Sci. 23:5. 10.1016/j.tplants.2018.02.005 29530380

[B37] Boroujeni S. M. Kalbasi M. Asgharzadeh A. Baharlouei J. (2021). Evaluating the potential of *Halothiobacillus* bacteria for sulfur oxidation and biomass production under saline soil. Geomicrobiol. J. 38, 57–65. 10.1080/01490451.2020.1809571

[B38] Brahmaprakash G. P. Sahu P. K. (2012). Biofertilizers for sustainability. J. Indian Institute Sci. 92, 37–62.

[B39] Cabral M. Cheng X. Singh S. Ivessa A. S. (2016). Absence of non-histone protein complexes at natural chromosomal pause sites results in reduced replication pausing in aging yeast cells. Cell Rep. 8, 1747–1754. 10.1016/j.celrep.2016.10.050 27829146 PMC5396545

[B40] Cataldo E. Fucile M. Mattii G. B. (2022). Biostimulants in viticulture: a sustainable approach against biotic and abiotic stresses. Plants 11:162. 10.3390/plants11020162 35050049 PMC8777853

[B41] Chandrasekaran M. Chanratana M. Kim K. Seshadri S. Sa T. (2019). Impact of arbuscular mycorrhizal fungi on photosynthesis, water status, and gas exchange of plants under salt stress–a meta-analysis. Front. Plant Sci. 10, 457. 10.3389/fpls.2019.00457 31040857 PMC6476944

[B42] Chaudhary A. Chaudhary P. Upadhyay A. Kumar A. Singh A. (2022a). Effect of gypsum on plant growth promoting rhizobacteria. Environ. Ecol. 39, 1248–1256.31124074

[B43] Chaudhary A. Parveen H. Chaudhary P. Khatoon H. Bhatt P. (2021). “Rhizospheric microbes and their mechanism,” in Microbial Technology for Sustainable Environment, eds P. Bhatt, S. Gangola, D. Udayanga, and G. Kumar (Singapore: Springer). 10.1007/978-981-16-3840-4_6

[B44] Chaudhary P. Chaudhary A. Agri U. Khatoon H. Singh A. (2022b). “Recent trends and advancements for agro-environmental sustainability at higher altitudes,” In: *Survival Strategies in Cold-adapted Microorganisms*, eds R. Goel, R. Soni, D. C. Suyal, and M. Khan M. (Singapore: Springer). 10.1007/978-981-16-2625-8_19

[B45] Chaudhary P. Chaudhary A. Bhatt P. Kumar G. Khatoon H. Rani A.. (2022c). Assessment of soil health indicators under the influence of nanocompounds and *Bacillus* spp. in field condition. Front. Environ. Sci. 9, 769871. 10.3389/fenvs.2021.769871

[B46] Chaudhary P. Sharma A. (2019). Response of nanogypsum on the performance of plant growth promotory bacteria recovered from nanocompound infested agriculture field. Environ. Ecol. 37, 363–372.

[B47] Checchio M. V. de Cássia Alves R. de Oliveira K. R. Moro G. V. Santos D. M. M. D. Gratão P. L. (2021). Enhancement of salt tolerance in corn using *Azospirillum brasilense*: An approach on antioxidant system. J. Plant Res. 24:34302571. 10.1007/s10265-021-01332-1 34302571

[B48] Chen Y. Yang H. Shen Z. Ye J. (2022). Whole-Genome Sequencing and Potassium-Solubilizing Mechanism of *Bacillus aryabhattai* SK1-7. Front. Microbiol. 12, 722379. 10.3389/fmicb.2021.722379 35058888 PMC8764406

[B49] Chen Z. Zhao L. Dong Y. Chen W. Li C. Gao X.. (2021). The antagonistic mechanism of *Bacillus velezensis* ZW10 against rice blast disease: Evaluation of ZW10 as a potential biopesticide. PLoS ONE 16, e0256807. 10.1371/journal.pone.0256807 34449822 PMC8396770

[B50] Cheng X. F. Wu H. H. Zou Y. N. Wu Q. S. Kuča K. (2021). Mycorrhizal response strategies of trifoliate orange under well-watered, salt stress, and waterlogging stress by regulating leaf aquaporin expression. Plant Physiol. Biochem. 162, 27–35. 10.1016/j.plaphy.2021.02.026 33662869

[B51] Chu T. N. Tran B. Van Bui L. Hoang M. (2019). Plant growth-promoting rhizobacterium *Pseudomonas* PS01 induces salt tolerance in *Arabidopsis thaliana*. BMC Res. Notes 12:11. 10.1186/s13104-019-4046-1 30635071 PMC6330407

[B52] Cissoko M. Hocher V. Gherbi H. Gully D. Carré-Mlouka A. Sane S.. (2018). Actinorhizal signaling molecules: *Frankia* root hair deforming factor shares properties with NIN inducing factor. Front. Plant Sci. 18:1494. 10.3389/fpls.2018.01494 30405656 PMC6201211

[B53] Collinge D. B. Jensen D. F. Rabiey M. Sarrocco S. Shaw M. W. Shaw R. H. (2022). Biological control of plant diseases – What has been achieved and what is the direction? Plant Pathol. 71, 1024–1047. 10.1111/ppa.13555

[B54] Dal C.ortivo C. Ferrari M. Visioli G. Lauro M. Fornasier F. Barion G.. (2020). Effects of seed-applied biofertilizers on rhizosphere biodiversity and growth of common wheat (*Triticum aestivum* L.) in the field. Front. Plant Sci. 11, 72. 10.3389/fpls.2020.00072 32174929 PMC7054350

[B55] Daroodi Z. Taheri P. S. (2021). Direct antagonistic activity and tomato resistance induction of the endophytic fungus *Acrophialophora jodhpurensis* against *Rhizoctonia solani*. Biol. Control. 160:104696. 10.1016/j.biocontrol.2021.104696

[B56] Desrut A. Moumen B. Thibault F. Le Hir R. Coutos-Thévenot P. Vriet C. (2020). Beneficial rhizobacteria *Pseudomonas simiae* WCS417 induce major transcriptional changes in plant sugar transport. J. Exp. Bot. 71, 7301–7315. 10.1093/jxb/eraa396 32860502

[B57] Dicko A. H. Nantoume D. Ouattara D. Kassogue A. Fane R. Malle I.. (2021). Screening of rice endophytic natives biofertilizers with plant growth-promoting characteristics. Afr. J. Agri. Res. 17, 1336–1342. 10.5897/AJAR2020.15191

[B58] Ding Z. Ali E. F. Almaroai Y. A. Eissa M. A. Abeed A. H. A. (2021). Effect of potassium solubilizing bacteria and humic acid on faba bean (*Vicia faba* L.) plants grown on sandy loam soils. J. Soil Sci. Plant Nutr. 21, 791–800. 10.1007/s42729-020-00401-z

[B59] Divekar P. A. Narayana S. Divekar B. A. Kumar R. Gadratagi B. G. Ray A.. (2022). Plant secondary metabolites as defense tools against herbivores for sustainable crop protection. Int. J. Mol. Sci. 23:2690. 10.3390/ijms23052690 35269836 PMC8910576

[B60] Don Y. Schmidtke S. M. Gambetta L. M. (2020). *Aureobasidium pullulans* volatilome identified by a novel, quantitative approach employing SPME-GC-MS, suppressed Botrytis cinerea and *Alternaria alternata in vitro*. Sci. Rep. 10:4498. 10.1038/s41598-020-61471-8 32161291 PMC7066187

[B61] dos Santos T. B Ribas A. F. de Souza S. G. H. Budzinski I. G. F. Domingues D.S. (2022). Physiological responses to drought, salinity, and heat stress in plants: a review. Stresses 2, 113–135. 10.3390/stresses2010009

[B62] Duan C. Mei Y. Wang Q. Wang Y. Li Q. Hong M.. (2022). Rhizobium inoculation enhances the resistance of alfalfa and microbial characteristics in copper-contaminated soil. Front. Microbiol. 12, 781831. 10.3389/fmicb.2021.781831 35095795 PMC8791600

[B63] Duc N. H. Csintalan Z. Posta K. (2018). Arbuscular mycorrhizal fungi mitigate negative effects of combined drought and heat stress on tomato plants. Plant Physiol. Biochem. 132, 297–307. 10.1016/j.plaphy.2018.09.011 30245343

[B64] Egamberdieva D. Alimov J. Shurigin V. Alaylar B. Wirth S. Bellingrath-Kimura S. D. (2022). Diversity and plant growth-promoting ability of endophytic, halotolerant bacteria associated with *Tetragonia tetragonioides* (Pall.) kuntze. Plants. 11:49. 10.3390/plants11010049 35009054 PMC8747539

[B65] Eichmann R. Richards L. Schafer P. (2021). Hormones as go-betweens in plant microbiome assembly. Plant J. 105, 518–541. 10.1111/tpj.15135 33332645 PMC8629125

[B66] Eid A. M. Fouda A. Abdel-Rahman M. A. Salem S. S. Elsaied A. Oelmüller R.. (2021). Harnessing bacterial endophytes for promotion of plant growth and biotechnological applications: an overview. Plants 10:935. 10.3390/plants10050935 34067154 PMC8151188

[B67] El-Shahir A. A. Noha A. El,-T Omar M. A. Arafat A. H. Abdel L.. (2021). The effect of endophytic *Talaromyces pinophilus* on growth, absorption and accumulation of heavy metals of *Triticum aestivum* grown on sandy soil amended by sewage sludge. Plants. 10:2659. 10.3390/plants10122659 34961130 PMC8704920

[B68] Esmaeel Q. Miotto L. Rondeau M. Leclère V. Clément C. Jacquard C. (2018). *Paraburkholderia phytofirmans* PsJN-plants interaction: from perception to the induced mechanisms. Front. Microbiol. 9, 2093. 10.3389/fmicb.2018.02093 30214441 PMC6125355

[B69] Etesami H. Jeong B. R. Glick B. R. (2021). Contribution of arbuscular mycorrhizal fungi, phosphate–solubilizing bacteria, and silicon to p uptake by plant. Front. Plant Sci. 12, 699618. 10.3389/fpls.2021.699618 34276750 PMC8280758

[B70] Etesami H. Mirseyed Hosseini H. Alikhani H. A. (2014). Bacterial biosynthesis of 1-aminocyclopropane-1-caboxylate (ACC) deaminase, a useful trait to elongation and endophytic colonization of the roots of rice under constant flooded conditions. Physiol. Mol. Biol. Plants. 20, 425–434. 10.1007/s12298-014-0251-5 25320466 PMC4185049

[B71] Fadiji A. E. Babalola O. O. (2020). Elucidating mechanisms of endophytes used in plant protection and other bioactivities with multifunctional prospects. Front. Bioeng. Biotechnol. 8:467. 10.3389/fbioe.2020.00467 32500068 PMC7242734

[B72] Fahsi N. Mahdi I. Mesfioui A. Biskri L. Allaoui A. (2021). Plant growth-promoting rhizobacteria isolated from the Jujube (*Ziziphus lotus*) plant enhance wheat growth, Zn uptake and heavy metal tolerance. Agriculture. 11:316. 10.3390/agriculture11040316

[B73] Fang L. Ju W. Yang C. Jin X. Zhang C. (2020). Exogenous application of signaling molecules to enhance the resistance of legume-rhizobium symbiosis in Pb/Cd-contaminated soils. Environ. Pollut. 365:114744. 10.1016/j.envpol.2020.114744 32806415

[B74] Fasusi O. A. Cruz C. Babalola O. O. (2021). Agricultural sustainability: microbial biofertilizers in rhizosphere management. Agriculture 11:163. 10.3390/agriculture11020163

[B75] Fortt J. González M. Morales P. Araya N. Remonsellez F. Coba de la Peña T.. (2022). Bacterial modulation of the plant ethylene signaling pathway improves tolerance to salt stress in lettuce (*Lactuca sativa* L.). Front. Sustain. Food Syst. 6:768250. 10.3389/fsufs.2022.768250

[B76] Galindo F. S. Pagliari P. H. Fernandes G. C. Rodrigues W. L. Boleta E. H. M. Jalal A.. (2022). Improving sustainable field-grown wheat production with *Azospirillum brasilense* under tropical conditions: a potential tool for improving nitrogen management. Front. Environ. Sci. 10, 821628. 10.3389/fenvs.2022.821628

[B77] Game B. C. Ilhe B. M. Pawar V. S. Khandagale P. P. (2020). Effect of *Azotobacter*, phosphate solubilizing bacteria and potash mobilizing bacteria inoculants on productivity of wheat (*Triticum aestivum* L.). Intern. J. Curr. Microbiol. Appl. Sci. 9, 2800–2807. 10.20546/ijcmas.2020.903.322

[B78] Gao D. Ran C. Zhang Y. Wang X. Lu S. Geng Y.. (2022). Effect of different concentrations of foliar iron fertilizer on chlorophyll fluorescence characteristics of iron-deficient rice seedlings under saline sodic conditions. Plant Physiol. Biochem. 185, 112–122. 10.1016/j.plaphy.2022.05.021 35671588

[B79] Ghaffari H. Gholizadeh A. Biabani A. Fallah A. Mohammadian M. (2018). Plant growth promoting rhizobacteria (PGPR) application with different nitrogen fertilizer levels in rice (*Oryza sativa* L.). Pertanika J. Trop. Agric. Sci. 41, 715–728.

[B80] Ghazy N. El-Nahrawy S. (2021). Siderophore production by *Bacillus subtilis* MF497446 and *Pseudomonas koreensis* MG209738 and their efficacy in controlling *Cephalosporium maydis* in maize plant. Arch. Microbiol. 203, 1195–1209. 10.1007/s00203-020-02113-5 33231747 PMC7683328

[B81] Ghodhbane-Gtari F. D'Angelo T. Gueddou A. Ghazouani S. Gtari M. Tisa L. S. (2021). Alone yet not alone: frankia lives under the same roof with other bacteria in actinorhizal nodules. Front. Microbiol. 12, 749760. 10.3389/fmicb.2021.749760 34925263 PMC8674757

[B82] Ghori N. H. Ghori T. Hayat M. Q. Imadi S. R. Gul A. Altay V.. (2019). Heavy metal stress and responses in plants. Int. J. Environ. Sci. Technol. 16, 1807–1828. 10.1007/s13762-019-02215-835205031

[B83] Ghosh D. Gupta A. Mohapatra S. (2019). A comparative analysis of exopolysaccharide and phytohormone secretions by four drought-tolerant rhizobacterial strains and their impact on osmotic-stress mitigation in *Arabidopsis thaliana*. World J. Microbiol. Biotechnol. 35, 1–15. 10.1007/s11274-019-2659-0 31147784

[B84] Gohil R. B. Raval V. H. Panchal R. R. Rajput K. N. (2022). Plant growth-promoting activity of Bacillus sp. PG-8 isolated from fermented panchagavya and its effect on the growth of arachis hypogea. Front. Agron. 4, 805454. 10.3389/fagro.2022.805454

[B85] Gond S. K. Torres M. S. Bergen M. S. Helsel Z. White J. F. (2015). Induction of salt tolerance and up-regulation of aquaporin genes in tropical corn by rhizobacterium *Pantoea agglomerans*. Lett. Appl. Microbiol. 60, 392–399. 10.1111/lam.12385 25557002

[B86] Gong Z. Xiong L. Shi H. Yang S. Herrera-Estrella L. R. Xu G.. (2020). Plant abiotic stress response and nutrient use efficiency. Sci. China Life Sci. 63, 635–674. 10.1007/s11427-020-1683-x 32246404

[B87] González-López M. D. C. Jijón-Moreno S. Dautt-Castro M. Ovando-Vázquez C. Ziv T. Horwitz B. A.. (2021). Secretome analysis of *Arabidopsis-Trichoderma atroviride* interaction unveils new roles for the plant glutamate:glyoxylate aminotransferase GGAT1 in plant growth induced by the fungus and resistance against botrytis cinerea. Int. J. Mol. Sci. 22:6804. 10.3390/ijms22136804 34202732 PMC8268252

[B88] González-Pérez E. Ortega-Amaro M. A. Salazar-Badillo F. B. Bautista E. Douterlungne D. Jiménez-Bremont J. F. (2018). The *Arabidopsis-Trichoderma* interaction reveals that the fungal growth medium is an important factor in plant growth induction. Sci. Rep. 8, 1–14. 10.1038/s41598-018-34500-w 30401880 PMC6219587

[B89] Gouda S. Kerry R. G. Das G. (2018). Revitalization of plant growth promoting rhizobacteria for sustainable development in agriculture. Microbiol. Res. 206, 131–140. 10.1016/j.micres.2017.08.016 29146250

[B90] Gu S. Wei Z. Shao Z. Friman V. P. Cao K. Yang T. (2020). Competition for iron drives phytopathogen control by natural rhizosphere microbiomes. Nat. Microbiol. 5, 1002–1010. 10.1038/s41564-020-0719-8 32393858 PMC7116525

[B91] Guo J. Chi J. (2014). Effect of Cd-tolerant plant growth promoting Rhizobium on plant growth and Cd uptake by *Lolium multiflorum* Lam. and Glycine max (L.) Merr. in Cd-contaminated soil. Plant Soil. 375, 205–214. 10.1007/s11104-013-1952-1

[B92] Gupta A. Rai S. Bano A. Khanam A. Sharma S. Pathak N. (2021). Comparative evaluation of different salt-tolerant plant growth-promoting bacterial isolates in mitigating the induced adverse effect of salinity in *pisum sativum*. Biointerface Res. Appl. Chem. 11, 13141–13154. 10.33263/BRIAC115.1314113154

[B93] Gupta S. Pandey S. (2019). ACC deaminase producing bacteria with multifarious plant growth promoting traits alleviates salinity stress in French bean (*Phaseolus vulgaris*) plants. Front. Microbiol. 10, 1506. 10.3389/fmicb.2019.01506 31338077 PMC6629829

[B94] Hao Z. Xie W. Jiang X. Wu Z. Zhang X. Chen B. (2019). Arbuscular mycorrhizal fungus improves *Rhizobium–Glycyrrhiza* seedling symbiosis under drought stress. Agronomy. 9:572. 10.3390/agronomy910057228424720

[B95] Harman G. Khadka R. Doni F. Uphoff N. (2020). Benefits to plant health and productivity from enhancing plant microbial symbionts. Front. Plant Sci. 11, 610065. 10.3389/fpls.2020.610065 33912198 PMC8072474

[B96] Haro R. Benito B. (2019). The role of soil fungi in K+ plant nutrition. Int. J. Mol. Sci. 20:3169. 10.3390/ijms20133169 31261721 PMC6651076

[B97] Hasan N. Farzand A. Heng Z. Khan I. U. Moosa A. Zubair M.. (2020). Antagonistic potential of novel endophytic *Bacillus* strains and mediation of plant defense against *Verticillium* wilt in upland cotton. Plants 9:1438. 10.3390/plants9111438 33113805 PMC7692591

[B98] Hassan E. A. Yasser S. Mostafa A. Mohamed H. Nivien A. N. (2021). Biosafe management of *Botrytis* grey mold of strawberry fruit by novel bioagents. Plants 12:2737. 10.3390/plants10122737 34961208 PMC8706406

[B99] Hata E. M. Yusof M. T. Zulperi D. (2021). Induction of systemic resistance against bacterial leaf streak disease and growth promotion in rice plant by *Streptomyces shenzhenesis* TKSC3 and *Streptomyces* sp. SS8. Plant Pathol. J. 37, 173–181. 10.5423/PPJ.OA.05.2020.0083 33866759 PMC8053841

[B100] Hawkins J. P. Oresnik I. J. (2022). The *Rhizobium*-legume symbiosis: co-opting successful stress management. Front. Plant Sci. 3, 796045. 10.3389/fpls.2021.796045 35046982 PMC8761673

[B101] He M. He C.-Q. Ding N.-Z. (2018). Abiotic stresses: general defenses of land plants and chances for engineering multistress tolerance. Front. Plant Sci. 9, 1771. 10.3389/fpls.2018.01771 30581446 PMC6292871

[B102] Hennessy L. M. Popay A. J. Glare T. R. (2022). Olfactory responses of Argentine stem weevil to herbivory and endophyte-colonisation in perennial ryegrass. J. Pest Sci. 95, 263–277. 10.1007/s10340-021-01375-2

[B103] Hernández-Fernández M. Cordero-Bueso G. Ruiz-Muñoz M. Cantoral J. M. (2021). Culturable yeasts as biofertilizers and biopesticides for a sustainable agriculture: a comprehensive review. Plants (Basel) 21:822. 10.3390/plants10050822 33919047 PMC8142971

[B104] Htwe A. Z. Moh S. M. Moe K. Yamakawa T. (2019). Biofertilizer production for agronomic application and evaluation of its symbiotic effectiveness in soybeans. Agronomy 9:162. 10.3390/agronomy9040162

[B105] Huang C. J. Wei G. Jie Y. C. Xu J. J. Zhao S. Y. Wang L. C.. (2015). Responses of gas exchange, chlorophyll synthesis and ROS-scavenging systems to salinity stress in two ramie (*Boehmeria nivea* L.) cultivars. Photosynthetica 53, 455–463. 10.1007/s11099-015-0127-0

[B106] Hummadi E. H. Cetin Y. Demirbek M. Kardar N. M. Khan S. Coates C. J.. (2022). Antimicrobial volatiles of the insect pathogen *Metarhizium brunneum*. J. Fungi 22:326. 10.3390/jof8040326 35448558 PMC9025432

[B107] Hussain A. Zahir Z. A. Ditta A. Tahir M. U. Ahmad M. Mumtaz M. Z.. (2020). Production and implication of bio-activated organic fertilizer enriched with zinc-solubilizing bacteria to boost up maize (*Zea mays* L.) production and biofortification under two cropping seasons. Agronomy 10:39. 10.3390/agronomy10010039

[B108] Igiehon N. O. Babalola O. O. Aremu B. R. (2019). Genomic insights into plant growth promoting rhizobia capable of enhancing soybean germination under drought stress. BMC Microbiol. 19:159. 10.1186/s12866-019-1536-1 31296165 PMC6624879

[B109] Igiehon N. O. Babalola O. O. Cheseto X. Torto B. (2021). Effects of rhizobia and arbuscular mycorrhizal fungi on yield, size distribution and fatty acid of soybean seeds grown under drought stress. Microbiol. Res. 242:126640. 10.1016/j.micres.2020.126640 33223380

[B110] Imran Q. M. Falak N. Hussain A. Mun B.-G. Yun B.-W. (2021). Abiotic stress in plants; stress perception to molecular response and role of biotechnological tools in stress resistance. Agronomy 11:1579. 10.3390/agronomy11081579

[B111] Iqbal Z. Iqbal M. S. Hashem A. Abd_Allah E. F. Ansari M. I. (2021). Plant defense responses to biotic stress and its interplay with fluctuating dark/light conditions. Front. Plant Sci. 12, 631810. 10.3389/fpls.2021.631810 33763093 PMC7982811

[B112] Ismail A. H. Mehmood A. Qadir M. Husna A. I. Hamayun M. Khan N. (2020). Thermal stress alleviating potential of endophytic fungus *Rhizopus oryzae* inoculated to sunflower (*Helianthus annuus* L.) and soybean (*Glycine max* L.). Pak. J. Bot. 52, 1857–1865. 10.30848/PJB2020-5(10)

[B113] Ismail E. G. Mohamed W. W. Khattab S. Sherif F. E. (2013). Effect of manure and bio-fertilizers on growth, yield, silymarin content, and protein expression profile of *Silybum marianum*. Int. J. Med. Arom. Plants. 3, 430–438.

[B114] Issa A. Esmaeel Q. Sanchez L. Courteaux B. Guise and, J.-F. Gibon Y. (2018). Impacts of *Paraburkholderia phytofirmans* strain PsJN on tomato (*Lycopersicon esculentum* L.) under high temperature. Front. Plant Sci. 9, 1397. 10.3389/fpls.2018.01397 30405648 PMC6201190

[B115] Jain D. Sharma J. Kaur G. Bhojiya A. A. Chauhan S. Sharma V.. (2021). Phenetic and molecular diversity of nitrogen-fixing plant growth-promoting *Azotobacter* isolated from semiarid regions of India. Hindawi BioMed. Res. Intern. 2021:6686283. 10.1155/2021/6686283

[B116] Janati W. Benmrid B. Elhaissoufi W. Zeroual Y. Nasielski J. Bargaz A. (2021). Will phosphate bio-solubilization stimulate biological nitrogen fixation in grain legumes? Front. Agron. 3:637196. 10.3389/fagro.2021.637196

[B117] Jiang W. Pan R. Wu C. Xu L. Abdelaziz M. E. Oelmüller R.. (2020). *Piriformospora indica* enhances freezing tolerance and post-thaw recovery in *Arabidopsi*s by stimulating the expression of CBF genes. Plant Signal. Behav. 15:1745472. 10.1080/15592324.2020.1745472 32228382 PMC7194378

[B118] Jimenez-Jimenez S. Santana O. Lara-Rojas F. Arthikala M. K. Armada E. Hashimoto K.. (2019). Differential *tetraspanin* genes expression and subcellular localization during mutualistic interactions in *Phaseolus vulgaris*. PLoS ONE 14, e0219765. 10.1371/journal.pone.0219765 31437164 PMC6705802

[B119] Jiménez-Mejía R. Medina-Estrada R. I. Carballar-Hernández S. Orozco-Mosqueda M. d,.C Santoyo G.. (2022). Teamwork to survive in hostile soils: use of plant growth-promoting bacteria to ameliorate soil salinity stress in crops. Microorganisms 10:150. 10.3390/microorganisms10010150 35056599 PMC8781547

[B120] Jing X. Cui Q. Li X. Yin J. Ravichandran V. Pan D.. (2020). Engineering *Pseudomonas protegens* Pf-5 to improve its antifungal activity and nitrogen fixation. Microb. Biotechnol. 13, 118–133. 10.1111/1751-7915.13335 30461205 PMC6984399

[B121] Kandel S. L. Joubert P. M. Doty S. L. (2017). Bacterial endophyte colonization and distribution within plants. Microorganisms 5:77. 10.3390/microorganisms5040077 29186821 PMC5748586

[B122] Kang S.-M. Radhakrishnan R. Khan A. L. Kim M.-J. Park J.-M. Kim B.-R. (2014b). Gibberellin secreting rhizobacterium, *Pseudomonas putida* H-2-3 modulates the hormonal and stress physiology of soybean to improve the plant growth under saline and drought conditions. Plant Physiol. Biochem. 84, 115–124. 10.1016/j.plaphy.2014.09.001 25270162

[B123] Kang S.-M. Shahzad R. Khan M. A. Hasnain Z. Lee K.-E. Park H.-S.. (2021). Ameliorative effect of indole-3-acetic acid- and siderophore-producing *Leclercia adecarboxylata* MO1 on cucumber plants under zinc stress. J. Plant Inter. 16, 30–41. 10.1080/17429145.2020.1864039

[B124] Kang S. M. Radhakrishnan R. You Y. H. Joo G. J. Lee I. J. Lee K. E.. (2014a). Phosphate solubilizing *Bacillus megaterium* mj1212 regulates endogenous plant carbohydrates and amino acids contents to promote mustard plant growth. Ind. J. Microbiol. 54, 427–433. 10.1007/s12088-014-0476-6 25320441 PMC4186932

[B125] Kaur S. Samota M. K. Choudhary M. Choudhary M. Pandey A. K. Sharma A.. (2022). How do plants defend themselves against pathogens-Biochemical mechanisms and genetic interventions. Physiol. Mol. Biol. Plants. 28, 485–504. 10.1007/s12298-022-01146-y 35400890 PMC8943088

[B126] Kenneth O. C. Nwadibe E. C. Kalu A. U. Unah U. V. (2019). Plant growth promoting rhizobacteria (PGPR): a novel agent for sustainable food production. Am. J. Agric. Biol. Sci. 14, 35–54. 10.3844/ajabssp.2019.35.54

[B127] Khan M. A. Asaf S. Khan A. L. Jan R. Kang S.-M. Kim K.-M. (2020). Extending thermotolerance to tomato seedlings by inoculation with SA1 isolate of *Bacillus cereus* and comparison with exogenous humic acid application. PLoS ONE 15, e0232228. 10.1371/journal.pone.0232228 32353077 PMC7192560

[B128] Khan N. Bano A. Zandi P. (2018). Effects of exogenously applied plant growth regulators in combination with PGPR on the physiology and root growth of chickpea (*Cicer arietinum*) and their role in drought tolerance. J. Plant Interact. 13, 239–247. 10.1080/17429145.2018.1471527

[B129] Khanna K. Jamwal V. L. Sharma A. Gandhi S. G. Ohri P. Bhardwaj R.. (2019). Supplementation with plant growth promoting rhizobacteria (PGPR) alleviates cadmium toxicity in *Solanum lycopersicum* by modulating the expression of secondary metabolites. Chemosphere. 230, 628–639. 10.1016/j.chemosphere.2019.05.072 31128509

[B130] Khare E. Mishra J. Arora N. K. (2018). Multifaceted interactions between endophytes and plant: developments and prospects. Front. Microbiol. 9, 2732. 10.3389/fmicb.2018.02732 30498482 PMC6249440

[B131] Khati P. Parul Bhatt P. Nisha Kumar R. Sharma A. (2018). Effect of nanozeolite and plant growth promoting rhizobacteria on maize. 3Biotech 8:141. 10.1007/s13205-018-1142-1 29484280 PMC5818361

[B132] Kirui C. K. Njeru E. M. Runo S. (2022). Diversity and phosphate solubilization efficiency of phosphate solubilizing bacteria isolated from semi-arid agroecosystems of Eastern Kenya. Microbiol. Insights 17:15. 10.1177/11786361221088991 35464120 PMC9019392

[B133] Köhl J. Kolnaar R. Ravensberg W. J. (2019). Mode of action of microbial biological control agents against plant diseases: relevance beyond efficacy. Front. Plant Sci. 10, 845. 10.3389/fpls.2019.00845 31379891 PMC6658832

[B134] Kousar B. Bano A. Khan N. (2020). PGPR Modulation of secondary metabolites in tomato infested with *Spodoptera litura*. Agronomy 10:778. 10.3390/agronomy10060778

[B135] Kukreti B. Sharma A. Chaudhary P. Agri U. Maithani D. (2020). Influence of nanosilicon dioxide along with bioinoculants on *Zea mays* and its rhizospheric soil. 3Biotech 10:345. 10.1007/s13205-020-02329-8 32728512 PMC7374527

[B136] Kumar A. Maurya B. M. Raghuwanshi R. (2021a). The microbial consortium of indigenous rhizobacteria improving plant health, yield and nutrient content in wheat (*Triticum aestivum*). *J. Plant Nutr*. 44, 1942–1956. 10.1080/01904167.2021.1884706

[B137] Kumar A. Singh S. K. Kant C. Verma H. Kumar D. Singh P. P.. (2021b). Microbial biosurfactant: a new frontier for sustainable agriculture and pharmaceutical industries. Antioxidants 10:1472. 10.3390/antiox10091472 34573103 PMC8469275

[B138] Kumar P. Dubey R. C. Maheshwari D. K. (2012). *Bacillus* strains isolated from rhizosphere showed plant growth promoting and antagonistic activity against phytopathogens. Microbiological Res. 16, 493–499. 10.1016/j.micres.2012.05.002 22677517

[B139] Kumar S. Kumar S. Mohapatra T. (2021c). Interaction between macro- and micro-nutrients in plants. Front. Plant Sci. 12, 665583. 10.3389/fpls.2021.665583 34040623 PMC8141648

[B140] Kusale S. P. Attar Y. C. Sayyed R. Z. Malek R. A. Ilyas N. Suriani N. L.. (2021). Production of plant beneficial and antioxidants metabolites by *Klebsiella variicola* under salinity stress. Molecules 26:1894. 10.3390/molecules26071894 33810565 PMC8037558

[B141] Lahlali R. Ezrari S. Radouane N. Kenfaoui J. Esmaeel Q. El Hamss H.. (2022). Biological control of plant pathogens: a global perspective. Microorganisms 10:596. 10.3390/microorganisms10030596 35336171 PMC8951280

[B142] Lata R. Chowdhury S. Gond S. K. White J.r, J.F. (2018). Induction of abiotic stress tolerance in plants by endophytic microbes. Lett. Appl. Microbiol. 66, 268–276. 10.1111/lam.12855 29359344

[B143] Lee L. H. Wu T. Y. Shak K. P. Y. Lim S. L. Ng K. Y. Nguyen M. N.. (2018). Sustainable approach to biotransform industrial sludge into organic fertilizer via vermicomposting: A mini-review. J. Chem. Technol. Biotechnol. 93, 925–935. 10.1002/jctb.5490

[B144] Liu H. Carvalhais L. C. Crawford M. Singh E. Dennis P. G. Pieterse C. M.. (2017a). Inner plant values: diversity, colonization and benefits from endophytic bacteria. Front. Microbiol. 8, 2552. 10.3389/fmicb.2017.02552 29312235 PMC5742157

[B145] Liu S. Tian Y. Jia M. Lu X. Yue L. Zhao X.. (2020). Induction of salt tolerance in *Arabidopsis thaliana* by volatiles from *Bacillus amyloliquefaciens* FZB42 via the jasmonic acid signaling patway. Front. Microbiol. 11, 562934. 10.3389/fmicb.2020.562934 33281760 PMC7688926

[B146] Liu Z. Rong Q. Zhou W. Liang G. (2017b). Effects of inorganic and organic amendment on soil chemical properties, enzyme activities, microbial community and soil quality in yellow clayey soil. PLoS ONE 12, e0172767. 10.1371/journal.pone.0172767 28263999 PMC5338777

[B147] Lopes M. J. S. Dias-Filho M. B. Gurgel E. S. C. (2021). Successful plant growth-promoting microbes: inoculation methods and abiotic factors. Front. Sustain. Food Syst. 5, 606454. 10.3389/fsufs.2021.606454

[B148] Lou X. Zhao J. Lou X. Xia X. Feng Y. Li H. (2022). The biodegradation of soil organic matter in soil-dwelling humivorous fauna. Front. Bioeng. Biotechnol. 9:808075. 10.3389/fbioe.2021.808075 35083207 PMC8784593

[B149] Luo J. Zhang Z. Hou Y. Diao F. Hao B. Bao Z.. (2021). Exploring microbial resource of different rhizocompartments of dominant plants along the salinity gradient around the hypersaline lake Ejinur. Front. Microbiol. 12, 698479. 10.3389/fmicb.2021.698479 34322109 PMC8312270

[B150] Lurthy T. Cantat C. Jeudy C. Declerck P. Gallardo K. Barraud C.. (2020). Impact of bacterial siderophores on iron status and ionome in pea. Front. Plant Sci. 11, 730. 10.3389/fpls.2020.00730 32595663 PMC7304161

[B151] Ma X. Liu Y. Shen W. Kuzyakov Y. (2021). Phosphatase activity and acidification in lupine and maize rhizosphere depend on phosphorus availability and root properties: coupling zymography with planar optodes. Agric. Ecosyst. Environ. Appl. Soil Ecol. 167:104029. 10.1016/j.apsoil.2021.104029

[B152] Mahanty T. Bhattacharjee S. Goswami M. Bhattacharyya P. Das B. Ghosh A.. (2017). Biofertilizers: a potential approach for sustainable agriculture development. Environ. Sci. Pollut. Res. 24, 3315–3335. 10.1007/s11356-016-8104-0 27888482

[B153] Mahmud A. A. Upadhyay S. K. Srivastava A. K. Bhojiya A. A. (2021). Biofertilizers: A Nexus between soil fertility and crop productivity under abiotic stress. Curr. Res. Environ. Sustain. 3:100063. 10.1016/j.crsust.2021.100063

[B154] Malik K. M. Khan K. S. Billah M. Akhtar M. S. Rukh S. Alam S.. (2021). Organic amendments and elemental sulfur stimulate microbial biomass and sulfur oxidation in alkaline subtropical soils. Agronomy 11:2514. 10.3390/agronomy11122514

[B155] Malusa E. Vassilev N. (2014). A contribution to set a legal framework for biofertilisers. Appl. Microbiol. Biotechnol. 98, 6599–6607. 10.1007/s00253-014-5828-y 24903811 PMC4108841

[B156] Marulanda A. Azcón R. Chaumont F. Ruiz-Lozano J. M. Aroca R. (2010). Regulation of plasma membrane aquaporins by inoculation with a *Bacillus megaterium* strain in maize (*Zea mays* L.) plants under unstressed and salt-stressed conditions. Planta 232, 533–543. 10.1007/s00425-010-1196-8 20499084

[B157] Mazid M. Khan T. A. (2015). Future of bio-fertilizers in Indian agriculture: an overview. Int. J. Agri. Food Res. 3:132. 10.24102/ijafr.v3i3.132

[B158] Meddich A. Ait M. M. Bourzik W. Mitsui T. Baslam M. Hafidi M. (2018). Optimizing Growth and Tolerance of Date Palm (Phoenix dactylifera L.) to Drought, Salinity, and Vascular Fusarium-Induced Wilt (Fusarium oxysporum) by Application of Arbuscular Mycorrhizal Fungi (AMF). Cham: Springer. 10.1007/978-3-319-75910-4_9

[B159] Meena K. K. Bitla U. M. Sorty A. M. Singh D. P. Gupta V. K. Wakchaure G. C.. (2020). Mitigation of salinity stress in wheat seedlings due to the application of phytohormone-rich culture filtrate extract of methylotrophic *Actinobacterium Nocardioides* sp. NIMMe6. Front. Microbiol. 11, 2091. 10.3389/fmicb.2020.02091 33071995 PMC7531191

[B160] Mesa-Marín J. Pérez-Romero J. A. Redondo-Gómez S. Pajuelo E. Rodríguez-Llorente I. D. Mateos-Naranjo E. (2020). Impact of plant growth promoting bacteria on *Salicornia ramosissima* ecophysiology and heavy metal phytoremediation capacity in estuarine soils. Front. Microbiol. 11, 553018. 10.3389/fmicb.2020.553018 33042058 PMC7527472

[B161] Mishra S. Arora N. K. (2012). Evaluation of rhizospheric *Pseudomonas* and *Bacillus* as biocontrol tool for *Xanthomonas campestris* pv campestris. World J. Microbiol. Biotechnol. 28, 693–702. 10.1007/s11274-011-0865-5 22806865

[B162] Mitter E. K. Tosi M. Obregón D. Dunfield K. E. Germida J. J. (2021). Rethinking crop nutrition in times of modern microbiology: innovative biofertilizer technologies. Front. Sustain. Food Syst. 5, 606815. 10.3389/fsufs.2021.606815

[B163] Mokabel S. Olama Z. Ali S. El-Dakak R. (2022). The role of plant growth promoting rhizosphere microbiome as alternative biofertilizer in boosting *Solanum melongena* L. Adaptation to salinity stress. Plants 11:659. 10.3390/plants11050659 35270129 PMC8912713

[B164] Mondal M. Skalicky M. Garai S. Hossain A. Sarkar S. H.. (2020). Supplementing nitrogen in combination with Rhizobium inoculation and soil mulch in peanut (*Arachis hypogaea* L.) production system: Part II. Effect on phenology, growth, yield attributes, pod quality, profitability and nitrogen use efficiency. Agronomy 10:1513. 10.3390/agronomy10101513

[B165] Morelli M. Bahar O. Papadopoulou K. K. Hopkins D. L. Obradovi,ć A. (2020). Role of endophytes in plant health and defense against pathogens. Front. Plant Sci. 11, 1312. 10.3389/fpls.2020.01312 32983202 PMC7479191

[B166] Morsy M. Blake C. Hayden A.-M. (2020). Fungal endophytes promote tomato growth and enhance drought and salt tolerance. Plants 9:7877. 10.3390/plants9070877 32664321 PMC7411952

[B167] Moustafa-Farag M. Almoneafy A. Mahmoud A. Elkelish A. Arnao M. B. Li L.. (2019). Melatonin and its protective role against biotic stress impacts on plants. Biomolecules 10:54. 10.3390/biom10010054 31905696 PMC7022677

[B168] Mukherjee A. Gaurav A. K. Singh S. Yadav S. Bhowmick S. Abeysinghe S.. (2022). The bioactive potential of phytohormones: A review. Biotechnol. Rep. 8:e00748. 10.1016/j.btre.2022.e00748 35719852 PMC9204661

[B169] Mukhtar S. Zareen M. Khaliq Z. Mehnaz S. Malik K. A. (2020a). Phylogenetic analysis of halophyte-associated rhizobacteria and effect of halotolerant and halophilic phosphate-solubilizing biofertilizers on maize growth under salinity stress conditions. J. Appl. Microbiol. 128, 556–573. 10.1111/jam.14497 31652362

[B170] Mukhtar T. Shafiqur R. Smith D. Sultan T. Seleiman M. F. Alsadon A. A.. (2020b). Mitigation of heat stress in *Solanum lycopersicum* L. by ACC-deaminase and exopolysaccharide producing Bacillus cereus: Effects on biochemical profiling. Sustainability 12:2159. 10.3390/su12062159

[B171] Nacoon S. Jogloy S. Riddech N. Mongkolthanaruk W. Kuyper T. Boonlue S. (2020). Interaction between phosphate solubilizing bacteria and arbuscular mycorrhizal fungi on growth promotion and tuber inulin content of *Helianthus tuberosus* L. Sci. Rep. 10:4916. 10.1038/s41598-020-61846-x 32188930 PMC7080738

[B172] Nandini B. Puttaswamy H. Saini R. K. Prakash H. S. Geetha N. (2021). Trichovariability in rhizosphere soil samples and their biocontrol potential against downy mildew pathogen in pearl millet. Sci. Rep. 11:9517. 10.1038/s41598-021-89061-2 33947949 PMC8096818

[B173] Nassal D. Spohn M. Eltlbany N. Jacquiod S. Smalla K. Marhan S.. (2018). Effects of phosphorus-mobilizing bacteria on tomato growth and soil microbial activity. Plant Soil. 427, 17–37. 10.1007/s11104-017-3528-y

[B174] Nie P. Chen C. Yin Q. Jiang C. Guo J. Zhao H.. (2019). Function of miR825 and miR825^*^ as negative regulators in *Bacillus cereus* AR156-elicited systemic resistance to *Botrytis cinerea* in *Arabidopsis thaliana*. Int. J. Mol. Sci. 20:5032. 10.3390/ijms20205032 31614458 PMC6829492

[B175] Nitu R. Rajinder K. Sukhminderjit K. (2020). Zinc solubilizing bacteria to augment soil fertility—A comprehensive review. Int. J. Agri. Sci. Vet. Med. 8, 38–44.

[B176] Niu J. Li X. (2022). Effects of microbial inoculation with different indigenous bacillus species on physicochemical characteristics and bacterial succession during short-term composting. Fermentation 8:152. 10.3390/fermentation8040152

[B177] Numan M. Bashir S. Khan Y. (2018). Plant growth promoting bacteria as an alternative strategy for salt tolerance in plants: a review. Microbiol Res. 209, 21–32. 10.1016/j.micres.2018.02.003 29580619

[B178] Odoh C. K. Sam K. Zabbey N. Eze C. N. Nwankwegu A. S. Laku C.. (2020). Microbial Consortium as Biofertilizers for Crops Growing Under the Extreme Habitats. Plant Microbiomes for Sustainable Agriculture. Cham: Springer. 10.1007/978-3-030-38453-1_13

[B179] Okamoto T. Shinjo R. Nishihara A. Uesaka K. Tanaka A. Sugiura D.. (2021). Genotypic variation of endophytic nitrogen-fixing activity and bacterial flora in rice stem based on sugar content. Front. Plant Sci. 2021, 1610. 10.3389/fpls.2021.719259 34447404 PMC8383490

[B180] Okur N. (2018). A review-bio-fertilizers-power of beneficial microorganisms in soils. Biomed. J. Sci. Tech. Res. 4, 4028–4029. 10.26717/BJSTR.2018.04.0001076

[B181] Oubohssaine M. Sbabou L. Aurag J. (2022). Native heavy metal-tolerant plant growth promoting rhizobacteria improves *Sulla spinosissima* (L.) growth in post-mining contaminated soils. Microorganisms 10:838. 10.3390/microorganisms10050838 35630284 PMC9144414

[B182] Oukala N. Aissat K. Pastor V. (2021). Bacterial endophytes: The hidden actor in plant immune responses against biotic stress. Plants 10:1012. 10.3390/plants10051012 34069509 PMC8161118

[B183] Pandey J. Singh A. (2012). Opportunities and constraints in organic farming: an Indian perspective. J. Sci. Res. 56, 47–72.

[B184] Pang Z. Chen J. Wang T. Gao C. Li Z. Guo L.. (2021). Linking plant secondary metabolites and plant microbiomes: a review. Front. Plant Sci. 12, 621276. 10.3389/fpls.2021.621276 33737943 PMC7961088

[B185] Paredes-Páliz K. Rodríguez-Vázquez R. Duarte B. Caviedes M. A. Mateos-Naranjo E. Redondo-Gómez S.. (2018). Investigating the mechanisms underlying phytoprotection by plant growth-promoting Rhizobacteria in *Spartina densiflora* under Metal Stress. Plant Biol. 20, 497–506 10.1111/plb.12693 29350476

[B186] Patel M. Patel K. Al-Keridis L. A. Alshammari N. Badraoui R. Elasbali A. M.. (2022). Cadmium-tolerant plant growth-promoting bacteria *Curtobacterium oceanosedimentum* improves growth attributes and strengthens antioxidant system in chili (*Capsicum frutescens*). Sustainability 14:4335. 10.3390/su14074335

[B187] Patel S. H. Viradiya M. B. Prajapati B. J. (2021). Effect of potassium and potassium mobilizing bacteria (KMB) with and without FYM on yield of wheat (*Triticum aestivum* L.). Pharmac. Phytochem. 10, 1615–1620.

[B188] Popp J. Pet,o K. Nagy J. (2013). Pesticide productivity and food security. A review. Agronomy Sustain. Dev. 33, 243–255. 10.1007/s13593-012-0105-x

[B189] Portieles R. Xu H. Yue Q. Zhao L. Zhang D. Du L.. (2021). Heat-killed endophytic bacterium induces robust plant defense responses against important pathogens. Sci. Rep. 11:12182. 10.1038/s41598-021-91837-5 34108579 PMC8190079

[B190] Qiang X. Ding J. Lin W. Li Q. Xu C. Zheng Q.. (2019). Alleviation of the detrimental effect of water deficit on wheat (*Triticum aestivum* L.) growth by an indole acetic acid-producing endophytic fungus. Plant Soil. 439, 373–391. 10.1007/s11104-019-04028-7

[B191] Qurashi A. W. Sabri A. N. (2012). Bacterial exopolysaccharide and biofilm formation stimulate chickpea growth and soil aggregation under salt stress. Braz. J. Microbiol. 43, 1183–1191. 10.1590/S1517-83822012000300046 24031943 PMC3768896

[B192] Rashid M. H. O. Khan A. Hossain M. T. Chung Y. R. (2017). Induction of systemic resistance against aphids by endophytic *Bacillus velezensis* YC7010 via expressing PHYTOALEXIN DEFICIENT4 in *Arabidopsis*. Front. Plant Sci. 8, 211. 10.3389/fpls.2017.00211 28261260 PMC5309228

[B193] Raza A. Razzaq A. Mehmood S. S. Zou X. Zhang X. Lv Y.. (2019). Impact of climate change on crops adaptation and strategies to tackle its outcome: A review. Plants 8, 34. 10.3390/plants8020034 30704089 PMC6409995

[B194] Reghmit A. Benzina-tihar F. Escudero F. J. L. Halouane-Sahir F. Oukali Z. Bensmail S.. (2021). *Trichoderma* spp. isolates from the rhizosphere of healthy olive trees in northern Algeria and their biocontrol potentials against the olive wilt pathogen, *Verticillium dahliae*. Org. Agric. 11, 639–657. 10.1007/s13165-021-00371-1

[B195] Rodriguez M. V. Tano J. Ansaldi N. Carrau A. Srebot M. S. Ferreira V.. (2019). Anatomical and biochemical changes induced by *Gluconacetobacter diazotrophicus* stand up for *Arabidopsis thaliana* seedlings from *Ralstonia solanacearum* infection. Front. Plant Sci. 10, 1618. 10.3389/fpls.2019.01618 31921261 PMC6936193

[B196] Romera F. J. García M. J. Lucena C. Martínez-Medina A. Aparicio M. A. Ramos J.. (2019). Induced systemic resistance (ISR) and Fe deficiency responses in dicot plants. Front. Plant Sci. 10, 287. 10.3389/fpls.2019.00287 30915094 PMC6421314

[B197] Roy S. Liu W. Nandety R. S. Crook A. Mysore K. S. Pislariu C. I.. (2020). Celebrating 20 years of genetic discoveries in legume nodulation and symbiotic nitrogen fixation. Plant Cell. 32, 15–41. 10.1105/tpc.19.00279 31649123 PMC6961631

[B198] Rybakova D. Rack-Wetzlinger U. Cernava T. Schaefer A. Schmuck M. Berg G. (2017). Aerial warfare: A volatile dialogue between the plant pathogen *Verticillium longisporum* and its antagonist *Paenibacillus polymyxa*. Front. Plant Sci. 8, 1294. 10.3389/fpls.2017.01294 28798756 PMC5529406

[B199] Safdarian M. Askari H. Shariati J. V. Nematzadeh G. (2019). Transcriptional responses of wheat roots inoculated with *Arthrobacter nitroguajacolicus* to salt stress. Sci. Rep. 9:1792. 10.1038/s41598-018-38398-2 30741989 PMC6370872

[B200] Salem M. Al-Amri (2021). Application of bio-fertilizers for enhancing growth and yield of common bean plants grown under water stress conditions. Saudi J. Biol. Sci. 28, 3901–3908. 10.1016/j.sjbs.2021.03.064 34220246 PMC8241702

[B201] Salomon M. V. Bottini R. de Souza Filho G. A. Cohen A. C. Moreno D. Gil M. (2014). Bacteria isolated from roots and rhizosphere of *Vitis vinifera* retard water losses, abscisic acid accumulation and synthesis of defence-related terpenes in in vitro cultured grapevine. Physiol. Plant. 151, 359–374. 10.1111/ppl.12117 24118032

[B202] Samain E. Aussenac T. Selim S. (2019). The effect of plant genotype, growth stage, and *Mycosphaerella graminicola* strains on the efficiency and durability of wheat-induced resistance by *Paenibacillus* sp. Strain B2. Front. Plant Sci. 10, 587. 10.3389/fpls.2019.00587 31143198 PMC6521617

[B203] Samaras A. Roumeliotis E. Ntasiou P. Karaoglanidis G. (2021). *Bacillus subtilis* MBI600 promotes growth of tomato plants and induces systemic resistance contributing to the control of soilborne pathogens. Plants 10:1113. 10.3390/plants10061113 34072940 PMC8229581

[B204] Sánchez-Montesinos B. Santos M. Moreno-Gavíra A. Marín-Rodulfo T. Gea F. J. Diánez F. (2021). Biological control of fungal diseases by T*richoderma aggressivum f. europaeum* and its compatibility with fungicides. J. Fungi. 24:7. 10.3390/jof7080598 34436137 PMC8397002

[B205] Sandhu N. Sethi M. Kumar A. Dang D. Singh J. Chhuneja P. (2021). Biochemical and genetic approaches improving nitrogen use efficiency in cereal crops: a review. Front. Plant Sci. 12, 657629. 10.3389/fpls.2021.657629 34149755 PMC8213353

[B206] Sandhya V. Ali S. Z. Grover M. Reddy G. Venkateswarlu B. (2010). Effect of plant growth promoting *Pseudomonas* spp. on compatible solutes, antioxidant status and plant growth of maize under drought stress. Plant Growth Regul. 62, 21–30. 10.1007/s10725-010-9479-4

[B207] Santosh S. Velmourougane K. Idapuganti R. G. Manikandan A. Blaise D. (2022). Potassium solubilizing potential of native bacterial isolates from cotton rhizosphere of rainfed vertisols. Natl. Acad. Sci. Lett. 2022, 1–4. 10.1007/s40009-022-01113-x

[B208] Sarkar J. Chakraborty B. Chakraborty U. (2018). Plant growth promoting rhizobacteria protect wheat plants against temperature stress through antioxidant signalling and reducing chloroplast and membrane injury. J. Plant Growth Regul. 37, 1396–1412. 10.1007/s00344-018-9789-8

[B209] Sarwar S. Khaliq A. Yousra M. Sultan T. Ahmad N. Khan M. Z. (2020). Screening of siderophore-producing PGPRs isolated from groundnut (*Arachis hypogaea* L.) rhizosphere and their influence on iron release in soil. Commun. Soil Sci. Plant Anal. 51, 1680–1692. 10.1080/00103624.2020.1791159

[B210] Sattar A. Naveed M. Ali M. Zahir Z. A. Nadeem S. M. Yaseen M.. (2019). Perspectives of potassium solubilizing microbes in sustainable food production system: A review. Agric,. Ecosyst. Environ,. Appl. Soil Ecol. 133, 146–159. 10.1016/j.apsoil.2018.09.012

[B211] Sdiri Y. Lopes T. Rodrigues N. Silva K. Rodrigues I. Pereira J. A.. (2022). Biocontrol ability and production of volatile organic compounds as a potential mechanism of action of olive endophytes against *Colletotrichum acutatum*. Microorganisms 10:571. 10.3390/microorganisms10030571 35336146 PMC8954755

[B212] Sendi Y. Pfeiffer T. Koch E. (2020). Potential of common bean (*Phaseolus vulgaris* L.) root microbiome in the biocontrol of root rot disease and traits of performance. J. Plant Dis. Prot. 127, 453–462 10.1007/s41348-020-00338-6

[B213] Sharma N. Khanna K. Manhas R. K. Bhardwaj R. Ohri P. Alkahtani J.. (2020). Insights into the role of *Streptomyces hydrogenans* as the plant growth promoter, photosynthetic pigment enhancer and biocontrol agent against *Meloidogyne incognita* in *Solanum lycopersicum* Seedlings. Plants 9:1109. 10.3390/plants9091109 32867342 PMC7570317

[B214] Sheteiwy M. S. Abd Elgawad H. Xiong Y.-C. Macovei A. Brestic M. Skalicky M. (2021) Inoculation with Bacillus amyloliquefaciens and mycorrhiza confers tolerance to drought stress improve seed yield quality of soybean plant. Physiologia Plant. 1–17. 10.1111/ppl.13454

[B215] Shukla P. S. Agarwal P. K. Jha B. I. (2012). Improved salinity tolerance of *Arachis hypogaea* (L.) by the interaction of halotolerant plant-growth-promoting rhizobacteria. J. Plant Growth Regul. 31, 195–206. 10.1007/s00344-011-9231-y

[B216] Shukla V. Kumar S. Tripathi Y. N. Upadhyay R. S. (2022). *Bacillus subtilis*- and *Pseudomonas fluorescens*-mediated systemic resistance in tomato against sclerotium rolfsii and study of physio-chemical alterations. Front. Fungal Biol. 3:851002. 10.3389/ffunb.2022.851002 PMC10512241 37746200

[B217] Singh P. Arif Y. Miszczuk E. Bajguz A. Hayat S. (2022a). Specific roles of lipoxygenases in development and responses to stress in plants. Plants 11:979. 10.3390/plants11070979 35406959 PMC9002551

[B218] Singh S. K. Wu X. Shao C. Zhang H. (2022b). Microbial enhancement of plant nutrient acquisition. Stress Biol. 2:3. 10.1007/s44154-021-00027-w PMC10441942 37676341

[B219] Soumare A. Diedhiou A. G. Thuita M. Hafidi M. Ouhdouch Y. Gopalakrishnan S.. (2020). Exploiting biological nitrogen fixation: a route towards a sustainable agriculture. Plants 9:1011. 10.3390/plants9081011 32796519 PMC7464700

[B220] Srivastava S. Verma P. C. Chaudhry V. Singh N. Abhilash P. C. Kumar K. V.. (2013). Influence of inoculation of arsenic-resistant *Staphylococcus arlettae* on growth and arsenic uptake in *Brassica juncea* (L.) Czern. Var. R-46. J. Hazard Mater. 15, 1039–1047. 10.1016/j.jhazmat.2012.08.019 22939092

[B221] Sujayanand G. K. Akram M. Konda A. Nigam A. Bhat S. Dubey J.. (2021). Distribution and toxicity of *Bacillus thuringiensis* (Berliner) strains from different crop rhizosphere in Indo-Gangetic plains against polyphagous lepidopteran pests. Int. J. Trop. Insect Sci. 41, 2713–2731. 10.1007/s42690-021-00451-5

[B222] Sultana S. Paul S. C. Parveen S. Alam S. Rahman N. Jannat B.. (2020). Isolation and identification of salt-tolerant plant-growth-promoting rhizobacteria and their application for rice cultivation under salt stress. Can. J. Microbiol. 66, 144–160. 10.1139/cjm-2019-0323 31714812

[B223] Sumbul A. Ansari R. A. Rizvi R. Mahmood I. (2020). *Azotobacter*: A potential bio-fertilizer for soil and plant health management. Saudi J. Biol. Sci. 27, 3634–3640. 10.1016/j.sjbs.2020.08.004 33304174 PMC7714982

[B224] Sundaramoorthy S. Balabaskar P. (2013). Evaluation of combined efficacy of *Pseudomonas fluorescens* and *Bacillus subtilis* in managing tomato wilt caused by *Fusarium oxysporum* f. sp. lycopersici (FOL). Plant Pathol. J. 12, 154–161. 10.3923/ppj.2013.154.161

[B225] Suzaki T. Takeda N. Nishida H. Hoshino M. Ito M. Misawa F.. (2019). Lack of symbiont accommodation controls intracellular symbiont accommodation in root nodule and arbuscular mycorrhizal symbiosis in *Lotus japonicus*. PLoS Genet. 15, e1007865. 10.1371/journal.pgen.1007865 30605473 PMC6317779

[B226] Taha R. S. Seleiman M. F. Shami A. Alhammad B. A. Mahdi A. H. A. (2021). Integrated application of selenium and silicon enhances growth and anatomical structure, antioxidant defense system and yield of wheat grown in salt-stressed soil. Plants. 10, 1040. 10.3390/plants10061040 34064224 PMC8224300

[B227] Tufail M. A. Touceda-González M. Pertot I. Ehlers R. U. (2021). *Gluconacetobacter diazotrophicus* Pal5 enhances plant robustness status under the combination of moderate drought and low nitrogen stress in *Zea mays* L. Microorganisms 9:870. 10.3390/microorganisms9040870 33920684 PMC8073419

[B228] Umair Hassan M. Aamer M. Umer Chattha M. Haiying T. Shahzad B. Barbanti L.. (2020). The critical role of zinc in plants facing the drought stress. Agriculture 10:396. 10.3390/agriculture10090396

[B229] Upadhyay S. K. Saxena A. K. Singh J. S. Singh D. P. (2019). Impact of native ST-PGPR (*Bacillus pumilus*; EU927414) on PGP traits, antioxidants activities, wheat plant growth and yield under salinity. Clim. Change Environ. Sustain. 7, 157–168. 10.5958/2320-642X.2019.00021.8

[B230] Usman M. Balsalobre-Lorente D. Jahanger A. Ahmad P. (2022). Pollution concern during globalization mode in financially resource-rich countries: Do financial development, natural resources, and renewable energy consumption matter? Renewable Ener. 183, 90–102. 10.1016/j.renene.2021.10.067

[B231] Vandana U. K. Rajkumari J. Singha L. P. Satish L. Alavilli H. Sudheer P. D. V. N.. (2021). The endophytic microbiome as a hotspot of synergistic interactions, with prospects of plant growth promotion. Biology 10:101. 10.3390/biology10020101 33535706 PMC7912845

[B232] Varga T. Hixson K. K. Ahkami A. H. Sher A. W. Barnes M. E. Chu R. K.. (2020). Endophyte-promoted phosphorus solubilization in populus. Front. Plant Sci. 11, 567918. 10.3389/fpls.2020.567918 33193494 PMC7609660

[B233] Vishwakarma K. Kumar N. Shandilya C. Mohapatra S. Bhayana S. Varma A. (2020). Revisiting plant–microbe interactions and microbial consortia application for enhancing sustainable agriculture: a review. Front. Microbiol. 11, 560406. 10.3389/fmicb.2020.560406 33408698 PMC7779480

[B234] Wang H. Wang Y. Jiang D. Xiang Z. Wang S. Kang C.. (2022a). Soil microbe inoculation alters the bacterial communities and promotes root growth of *Atractylodes lancea* under heat stress. *Plant Soil*. 10.1007/s11104-022-05369-6

[B235] Wang R. Lin J.-Q. Liu X.-M. Pang X. Zhang C.-J. Yang C.-L.. (2019). Sulfur oxidation in the acidophilic autotrophic *Acidithiobacillus* spp. Front. Microbiol. 9, 3290. 10.3389/fmicb.2018.03290 30687275 PMC6335251

[B236] Wang X. Cai D. Ji M. Chen Z. Yao L. Han H. (2022b). Isolation of heavy metal-immobilizing and plant growth-promoting bacteria and their potential in reducing Cd and Pb uptake in water spinach. Sci. Total Environ. 819:153242. 10.1016/j.scitotenv.2022.153242 35051479

[B237] Wang Y. Yang Z. Kong Y. Li X. Li W. Du H.. (2020). GmPAP12 is required for nodule development and nitrogen fixation under phosphorus starvation in soybean. Front. Plant Sci. 11, 450. 10.3389/fpls.2020.00450 32499790 PMC7243344

[B238] Wani P. A. Khan M. S. (2013). Nickel detoxification and plant growth promotion by multi metal resistant plant growth promoting *Rhizobium* species RL9. Bull. Environ. Contam. Toxicol. 91, 117–124. 10.1007/s00128-013-1002-y 23609454

[B239] Waqas M. Khan A. L. Kamran M. Hamayun M. Kang S. M. Kim Y. H.. (2012). Endophytic fungi produce gibberellins and indoleacetic acid and promotes host-plant growth during stress. Molecules 17, 10754–10773. 10.3390/molecules170910754 22960869 PMC6268353

[B240] Win T. T. Bo B. Malec P. Khan S. Fu P. (2021). Newly isolated strain of *Trichoderma asperellum* from disease suppressive soil is a potential bio-control agent to suppress Fusarium soil borne fungal phytopathogens. J. Plant Pathol. 103, 549–561. 10.1007/s42161-021-00780-x

[B241] Wu W. Du K. Kang X. Wei H. (2021a). The diverse roles of cytokinins in regulating leaf development. Hortic Res. 8:118. 10.1038/s41438-021-00558-3 34059666 PMC8167137

[B242] Wu Y. Huang W. Tian Q. Liu J. Xia X. Yang X.. (2021b). Comparative transcriptomic analysis reveals the cold acclimation during chilling stress in sensitive and resistant passion fruit (*Passiflora edulis*) cultivars. Peer J. 9:e10977. 10.7717/peerj.10977 33717701 PMC7936571

[B243] Xia Y. Liu J. Chen C. Mo X. Tan Q. He Y.. (2022). The multifunctions and future prospects of endophytes and their metabolites in plant disease management. Microorganisms 10:1072. 10.3390/microorganisms10051072 35630514 PMC9146654

[B244] Yadav A. N. Verma P. Kumar S. Kumar V. Kumar M. Kumari Sugitha T. C. (2018). Actinobacteria From Rhizosphere in New and Future Developments in Microbial Biotechnology and Bioengineering. 10.1016/B978-0-444-63994-3.00002-3

[B245] Yang Y. Chen Y. Cai J. Liu X. Huang G. (2021). Antifungal activity of volatile compounds generated by endophytic fungis *Sarocladium brachiariae* HND5 against *Fusarium oxysporum* f. sp. cubense. PLoS ONE 16, e0260747. 10.1371/journal.pone.0260747 34855862 PMC8639089

[B246] Yasmin H. Naeem S. Bakhtawar M. Jabeen Z. Nosheen A. Naz R. (2020). Halotolerant rhizobacteria *Pseudomonas pseudoalcaligenes* and *Bacillus subtilis* mediate systemic tolerance in hydroponically grown soybean (*Glycine max* L.) against salinity stress. PLoS ONE. 15, e0231348. 10.1371/journal.pone.0231348 32298338 PMC7162512

[B247] Yasuda M. Dastogeer K. M. G. Sarkodee-Addo E. Tokiwa C. Isawa T. Shinozaki S.. (2022). Impact of *Azospirillum* sp. B510 on the rhizosphere microbiome of rice under field conditions. Agronomy 12:1367. 10.3390/agronomy12061367

[B248] You J. M. Xiong K. Mu S. Guo J. Guo X. L. Duan Y. Y.. (2018). Identification of endophytic bacteria Bzjn1 and research on biological control of root rot of *Atractylodes Macrocephala*. Zhongguo Zhong Yao Za Zhi. 3, 478–483. 10.19540/j.cnki.cjcmm.20180105.008 29600611

[B249] Yu C. Fan L. Gao J. Wang M. Wu Q. Tang J.. (2015). The platelet-activating factor acetyl hydrolase gene derived from *Trichoderma harzianum* induces maize resistance to *Curvularia lunata* through the jasmonic acid signaling pathway. J. Environ. Sci. Health B. 50, 708–717. 10.1080/03601234.2015.1048104 26273755

[B250] Yu Y. Gui Y. Li Z. Jiang C. Guo J. Niu D. (2022). Induced systemic resistance for improving plant immunity by beneficial microbes. Plants 11:386. 10.3390/plants11030386 35161366 PMC8839143

[B251] Yu Z. Wang Z. Zhang Y. Wang Y. Liu Z. (2021). Biocontrol and growth-promoting effect of *Trichoderma asperellum* TaspHu1 isolate from *Juglans mandshurica* rhizosphere soil. Microbiol. Res. 242:e126596. 10.1016/j.micres.2020.126596 33007636

[B252] Zain M. Yasmin S. Hafeez F. Y. (2019). Isolation and characterization of plant growth promoting antagonistic bacteria from cotton and sugarcane plants for suppression of phytopathogenic *Fusarium* species. Iran. J. Biotechnol. 17:e1974. 10.21859/ijb.1974 31457052 PMC6697841

[B253] Zhang L. Xu M. Liu Y. Zhang F. Hodge A. Feng G. (2016). Carbon and phosphorus exchange may enable cooperation between an arbuscular mycorrhizal fungus and a phosphate solubilizing bacterium. New Phytol. 210, 1022–1032. 10.1111/nph.13838 27074400

[B254] Zhang T. Hu F. Ma L. (2019). Phosphate-solubilizing bacteria from safflower rhizosphere and their effect on seedling growth. Open. Life Sci. 14, 246–254. 10.1515/biol-2019-0028 33817158 PMC7874793

[B255] Zhang X. Liu Z. Wei G. Yang F. Liu X. (2018). In silico genome-wide analysis reveals the potential links between core genome of *Acidithiobacillus thiooxidans* and its autotrophic lifestyle. Front. Microbiol. 9, 1255. 10.3389/fmicb.2018.01255 29937764 PMC6002666

[B256] Zhou C. Zhu L. Xie Y. Li F. Xiao X. Ma Z.. (2017). *Bacillus licheniformis* SA03 confers increased saline–alkaline tolerance in *Chrysanthemum* plants by induction of abscisic acid accumulation. Front. Plant Sci. 8, 1143. 10.3389/fpls.2017.01143 28706529 PMC5489591

[B257] Zhou X. R. Dai L. Xu G. F. Wang H. S. (2021). A strain of *Phoma* species improves drought tolerance of *Pinus tabulaeformis*. Sci. Rep. 11:7637. 10.1038/s41598-021-87105-1 33828138 PMC8027514

[B258] Zhu L. Wang Y. Lu J. Liu S. Min Y. Liu X.. (2021). Complete genome sequence of *Bacillus badius* NBPM-293, a plant growth-promoting strain isolated from rhizosphere soil. Am. Soc. Microbiol. 10, e00977–e00921. 10.1128/MRA.00977-21 34761955 PMC8582306

[B259] Zulfikar S. A. Sandhya V. Grover M. Linga V. R. Bandi V. (2011). Effect of inoculation with a thermotolerant plant growth promoting Pseudomonas putida strain AKMP7 on growth of wheat (Triticum spp.) under heat stress, J. Plant Inter. 6, 239–246. 10.1080/17429145.2010.545147

